# Activatable polymer nanoagonist for second near-infrared photothermal immunotherapy of cancer

**DOI:** 10.1038/s41467-021-21047-0

**Published:** 2021-02-02

**Authors:** Yuyan Jiang, Jiaguo Huang, Cheng Xu, Kanyi Pu

**Affiliations:** 1 School of Chemical and Biomedical Engineering, Nanyang Technological University, Singapore, Singapore; 2 Division of Chemistry and Biological Chemistry, School of Physical and Mathematical Sciences, Nanyang Technological University, Singapore, Singapore

**Keywords:** Drug delivery, Nanotechnology in cancer

## Abstract

Nanomedicine in combination with immunotherapy offers opportunities to treat cancer in a safe and effective manner; however, remote control of immune response with spatiotemporal precision remains challenging. We herein report a photothermally activatable polymeric pro-nanoagonist (APNA) that is specifically regulated by deep-tissue-penetrating second near-infrared (NIR-II) light for combinational photothermal immunotherapy. APNA is constructed from covalent conjugation of an immunostimulant onto a NIR-II semiconducting transducer through a labile thermo-responsive linker. Upon NIR-II photoirradiation, APNA mediates photothermal effect, which not only triggers tumor ablation and immunogenic cell death but also initiates the cleavage of thermolabile linker to liberate caged agonist for in-situ immune activation in deep solid tumor (8 mm). Such controlled immune regulation potentiates systemic antitumor immunity, leading to promoted cytotoxic T lymphocytes and helper T cell infiltration in distal tumor, lung and liver to inhibit cancer metastasis. Thereby, the present work illustrates a generic strategy to prepare pro-immunostimulants for spatiotemporal regulation of cancer nano-immunotherapy.

## Abstract (web summary)

Precise control of immune response remains challenging for cancer immunotherapy. Here, the authors report on photothermally activatable semiconducting polymeric pro-agonist in response to second near-infrared window light for regulated photothermal immunotherapy.

## Introduction

Immunotherapy that boosts host immune system to fight against tumor has revolutionized cancer treatment^1,2^. However, single model immunotherapy often suffers from limited response rate and occasional immune-related adverse effects (irAEs) such as cytokine storm, hematopoietic system dysfunction, and organ failure^3^. Nanomedicine thus has been brought into cancer immunotherapy in the hope to address these critical concerns in view of its tunable physicochemical properties to reinforce drug–immune cell interactions, and optimal pharmacological profiles (e.g., biodistribution and retention) of immunotherapeutic molecules^4–6^. More importantly, nanomedicine serves as an assembly platform to allow additional therapeutic modalities (e.g., chemotherapy, phototherapy, radiotherapy, etc.) to be seamlessly integrated with immunotherapy to enhance therapeutic efficacy^7–11^. With the promise to improve immune response at no expense of breaking the systemic immune tolerance, cancer nano-immunotherapy has been recently under extensive development.

Further enhancement in the specificity of cancer immunotherapy to facilitate its clinical translation mainly lies in the development of smart immunotherapeutic nanoagents with controlled activation^12–14^. Till now, immunotherapeutic nanoagents have been programmed to unleash therapeutic action in response to biochemical indexes or cancer biomarkers such as pH, reactive oxidative species, and enzymes^15–18^. For instance, human interleukin-15 super-agonist complex was engineered with disulfide-containing cross-linker for activatable release in response to T-cell receptor activation so as to improve therapeutic window of adjuvant cytokine therapy^19^. As another example, programmed cell death protein 1 antibody was specifically conjugated to magnetic nanoclusters via pH-responsive benzoic-imine bond, leading to selective activation in the acidic intratumoral microenvironment for potentiation of adoptive T-cell therapy^20^. However, owing to their reliance on the difference of biomarkers at basal and pathological levels, the bioavailability and regional selectivity of immune activation for such immunotherapeutic nanoagents remain to be improved.

In contrast to endogenous biomarkers, external stimuli are independent of the physiological environment and thus hold potential for more precise spatiotemporal and dosage control over immune regulation^12,21^. Near-infrared light (NIR-I, 650–950 nm) is a popular external stimulus for the construction of smart nanomedicine owing to its minimal invasiveness and easy operation^22^. In particular, NIR-I light-responsive nanoagents have been developed to serve as the signal transducers to convert incident photons into regulatory signals, such as singlet oxygen to trigger the activation of indoleamine 2,3-dioxygenase 1 inhibitor, or high-energy emission to liberate CpG^21,23^. Superior to NIR-I light, second NIR light (NIR-II, 1000–1300 nm) has been recently revealed to possess even better biological transparency and further ameliorated phototoxicity with a lower maximum permissible exposure limit^24^. However, few NIR-II nanotransducers are available, which include gold nanostructures^25^, transition metal-based nanoparticles^26^, and naphthalocyanine derivatives^27^. Recently, semiconducting polymer nanoparticles (SPNs) composed of highly π-conjugated backbones have formed a promising class of NIR-II nanoagents^28,29^. With their excellent photothermal performance and intrinsically benign compositions, SPNs have been exploited for deep-tissue molecular photoacoustic imaging^30,31^, photothermal therapy (PTT)^32^, and photothermal ferrotherapy^33^. However, the integration of SPNs with NIR-II light for spatiotemporal photoregulation of immunotherapy has yet to be explored.

**Fig. 1:**
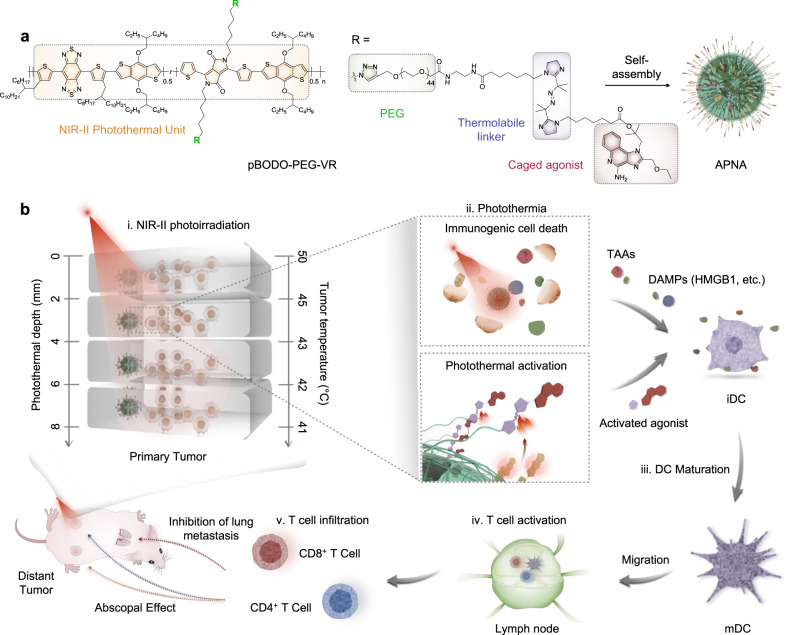
Scheme of APNA-mediated NIR-II photothermal immunotherapy. **a** Chemical structure of pBODO-PEG-VR and preparation of APNA. **b** Mechanism of antitumor immune response by APNA-mediated NIR-II photothermal immunotherapy. *TAAs* tumor-associated antigens, *DAMPs* damage-associated molecular patterns, *iDC* immature DC, *mDC* mature DC, *HMGB1* high-mobility group box 1 protein.

We herein report the synthesis of an activatable polymer nanoagonist (APNA) for NIR-II light-regulated photothermal immunotherapy of cancer (Fig. 1). APNA is composed of a NIR-II light-absorbing semiconducting polymer backbone as photothermal transducer, conjugated with a potent toll-like receptor type 7 and 8 (TLR7/8) agonist (Resiquimod: R848) as the immunostimulant through a thermolabile cleavable linker (2,2′-azobis[2-(2-imidazolin-2-yl)propane]: VA-044) (Fig. 1a). Mainly acting on antigen-presenting cells (APCs) such as dendritic cells (DCs), R848 helps upregulate secretion of crucial proinflammatory cytokines and enhance maturation or polarization of APCs, priming T lymphocytes^34^. Upon focal NIR-II photoirradiation, APNA mediates photothermal effect to directly ablate tumor, and elicit immunogenic cell deaths (ICDs) of cancer cells to promote antitumor immunity; it also in situ triggers the cleavage of thermolabile linker at the tumor site to activate the TLR7/8 agonist so as to further potentiate antitumor immune response (Fig. 1b). Such an APC-mediated spatiotemporal potentiation of cancer immunotherapy enables complete eradication of the primary tumor in deep tissue (~8 mm) and efficient inhibition of both distal tumor and lung metastasis without eliciting obvious systemic adverse effects. Furthermore, the underlying mechanisms of photothermal activation of immune response along the gradient of photothermal depth and in situ photothermal temperature in the deep beds of tumor are unveiled, providing guidelines for the development of photothermal immunotherapy.

## Results

### Synthesis and in vitro characterization

**Fig. 2:**
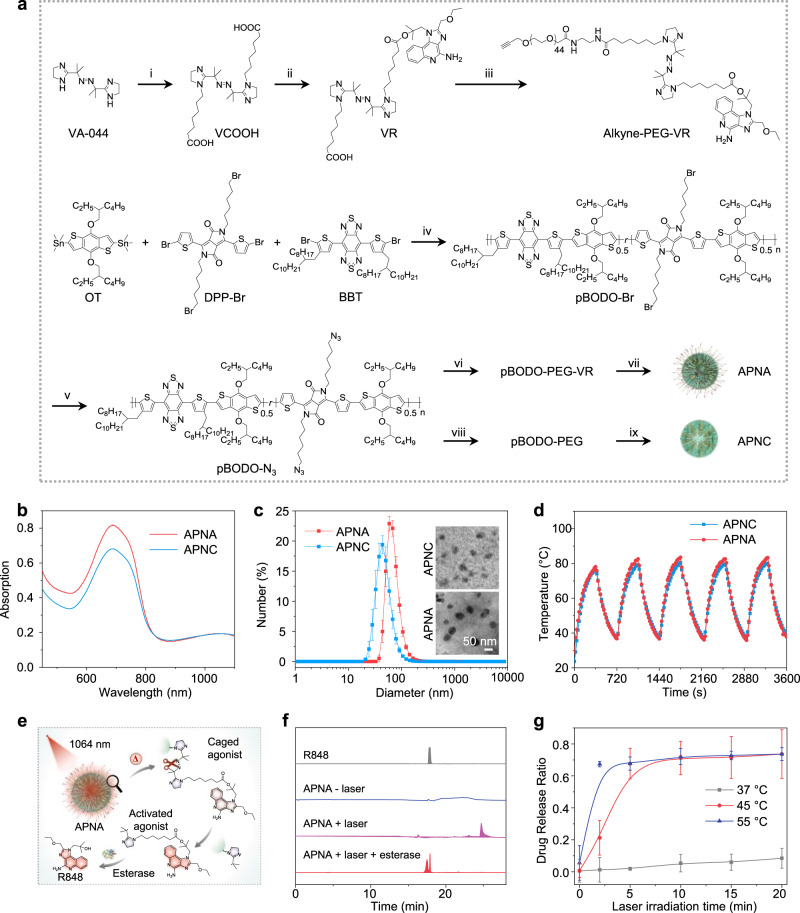
In vitro characterization of APNA. **a** Synthetic routes of APNA and APNC. (i) 7-iodoheptanoic acid, sodium hydride (NaH), dry tetrahydrofuran (THF), 25 °C, 2d. (ii) R848, 1-ethyl-3-(3-dimethylaminopropyl)carbodiimide (EDC), 4-dimethylaminopyridine (DMAP), dry acetonitrile (ACN), 25 °C, 3 d. (iii) alkyne-PEG-NH_2_, EDC, *N*-hydroxysuccinimide (NHS), dry THF, 25 °C, 2d. (iv) Pd_2_(dba)_3_, tri(*o*-tolyl)phosphine, chlorobenzene, 100 °C, 2 h. (v) sodium azide, dimethylformamide (DMF)/THF, 25 °C, 2d. (vi) alkyne-PEG-VR, CuBr, *N,N,N’,N”,N”*-pentamethyldiethylenetriamine (PMDETA), dry THF, 25 °C, 2d. (vii) aqueous self-assembly of pBODO-PEG-VR. (viii) methoxy-PEG-alkyne, CuBr, PMDETA, dry THF, 25 °C, 2d. (ix) aqueous self-assembly of pBODO-PEG. **b** Absorption spectra of APNA and APNC in 1× PBS solution. **c** DLS profiles of APNA and APNC nanoparticles. Inset: TEM images of APNA and APNC nanoparticles. **d** Photostability studies of APNA and APNC ([pBODO] = 20 µg mL^−1^) by photothermal heating (1064 nm photoirradiation, 1 W cm^−2^, 6 min) and cooling (natural cooling, 6 min) cycles. **e** Schematic illustration of photothermally triggered agonist release from APNA and subsequent hydrolysis by esterase. **f** HPLC analysis of photothermally triggered agonist release from APNA ([pBODO] = 50 µg mL^−1^). 1064 nm photoirradiation: 1 W cm^−2^, 10 min. **g** Relationship of photothermal activation ratio and different photothermal temperatures ([pBODO] = 50 µg mL^−1^). The photothermal temperature was controlled by switching power density of photoirradiation. Data were expressed as mean ± SD. Error bars indicated standard deviations of three independent measurements.

To obtain the NIR-II-absorbing semiconducting polymer precursor pBODO-Br, Stille polycondensation was used to copolymerize three monomers: 4,8-bis[5-bromo-4-(2-octyldodecyl)-2-thienyl]-benzo[1,2c:4,5c′]bis[1,2,5]thiadiazole (BBT), 2,5-bis(6-bromohexyl)-3,6-bis(5-bromothiophen-2-yl)pyrrolo[3,4-c]pyrrole-1,4(2H,5H)-dione (DPP-Br), and (4,8-bis((2-ethylhexyl)oxy)benzo[1,2b:4,5-b′]dithiophene-2,6-diyl)bis(trimethylstannane) (OT) (Fig. 2a). Because of the strong-electron withdrawing of BBT and the electron-rich OT, pBODO-Br has a charge transfer backbone and narrowed band gap, showing strong absorption in the NIR-II window (Supplementary Fig. 1). Gel permeation chromatography analysis indicated that pBODO-Br had a number average molecular weight (Mn) of 9826 (Supplementary Fig. 2). pBODO-Br was further transformed to pBODO-N_3_ through substitution of bromide with azide for post-functionalization (Supplementary Fig. 3). To conjugate the thermo-responsive linker VA-044 with the immuno-agonist R848, VA-044 was first modified with 7-iodoheptanoic acid, followed by Steglich esterification reaction with R848 to afford caged agonist VR (Supplementary Figs. 4, 5). A bifunctional poly(ethylene glycol) (PEG, Mw = 2000) with one propargyl terminal and the other amine terminal (alkyne-PEG-NH_2_) was synthesized and linked to VR via EDC/NHS coupling to obtain alkyne-PEG-VR conjugate (Supplementary Figs. 6, 7). Thereafter, pBODO-N_3_ was grafted with alkyne-PEG-VR to afford semiconducting pro-agonist pBODO-PEG-VR through click reaction (Supplementary Fig. 8). The amphiphilicity of pBODO-PEG-VR allowed its spontaneous assembly in aqueous solution to form nanoparticles termed as APNA (Fig. 1a). To prepare the control nanoparticle without pro-agonist termed as APNC, pBODO-N_3_ was conjugated with alkyne terminated PEG (pBODO-PEG), followed by self-assembly in aqueous solution.

Optical and colloidal properties of APNA were studied and compared with APNC. Both APNA and APNC had similar absorption spectra with maxima at 690 nm in NIR-I window and 1060 nm in NIR-II window (Fig. 2b), showing that conjugation of pro-agonist had negligible influence on NIR-II light-harvesting property. Whereas dynamic light scattering (DLS) indicated a larger hydrodynamic size of APNA (71 nm) than APNC (48 nm) (Fig. 2c), probably owing to the presence of hydrophobic agonist molecules. Transmission electron microscopy (TEM) further confirmed spherical morphology for both APNA and APNC (Fig. 2c). In addition, zeta potential measurement indicated a relatively neutralized surface charge of APNA (−13 mV) in contrast with APNC (−29 mV) (Supplementary Fig. 9a). Both nanoparticles showed negligible size change during storage in aqueous solutions for 2 months (Supplementary Fig. 9b), suggesting their excellent colloidal stability. According to high performance liquid chromatography (HPLC) calibration curve, the drug loading capacity of APNA was ca. 5.3%, suggesting that each pBODO-PEG-VR molecule contained ~3.3 equivalents of R848 pro-drug.

Photothermal property of APNA was evaluated and compared with APNC. Upon continuous NIR-II (1064 nm) photoirradiation, both APNA and APNC induced significant temperature rise of aqueous solution (Fig. 2d). After irradiation for 6 min, the maximum solution temperatures of APNA and APNC nanoparticles were 78 and 76 °C, respectively. Such phenomenon implied the negligible impact of agonist conjugation on the photothermal transduction capability of semiconducting backbone. Indeed, APNA and APNC had similar photothermal conversion efficiency as high as 84.4% at 1064 nm (Supplementary Fig. 10). In addition, negligible changes in maximal solution temperatures were observed for both APNA and APNC nanoparticles throughout five heating and natural cooling cycles, suggesting their excellent photostability as photothermal agents.

Photothermal activation of pro-agonist from APNA was evaluated and analyzed by HPLC (Fig. 2e, f). Without photoirradiation, no elution peak related to free R848 (*T*_R_ = 17.9 min) from APNA nanoparticles could be measured (Fig. 2f). After continuous photoirradiation of APNA solution for 10 min, an elution peak at 24.7 min assigned to the released agonist was observed. Such product from the photothermal cleavage was further confirmed by liquid chromatography-mass spectrometry (LCMS) and proton nuclear magnetic resonance spectroscopy (^1^H NMR) (Supplementary Figs. 11, 12). Through subsequent hydrolysis by esterase, the parent R848 was able to be liberated from the photothermally activated pro-agonist in an intact form, which was validated by both HPLC profiles and LCMS (Fig. 2f, Supplementary Fig. 13). Thereafter, the relationship between photothermal temperature and photothermal activation rate was investigated (Fig. 2g). When the photothermal temperature was kept at 37 °C for 20 min under photoirradiation, a maximal agonist activation ratio of 8.5% was achieved. Whereas such ratio was dramatically promoted to 74% when photothermal temperature was elevated to 45 °C or 55 °C and maintained for 20 min. Notably, such high activation ratio was achieved in 5 min with photothermal temperature at 55 °C, whereas extended to 10 min at 45 °C. These results indicated the agonist could be selectively activated by photothermal heating mediated by the backbone of APNA.

### In vitro photothermal ablation and DC maturation

**Fig. 3:**
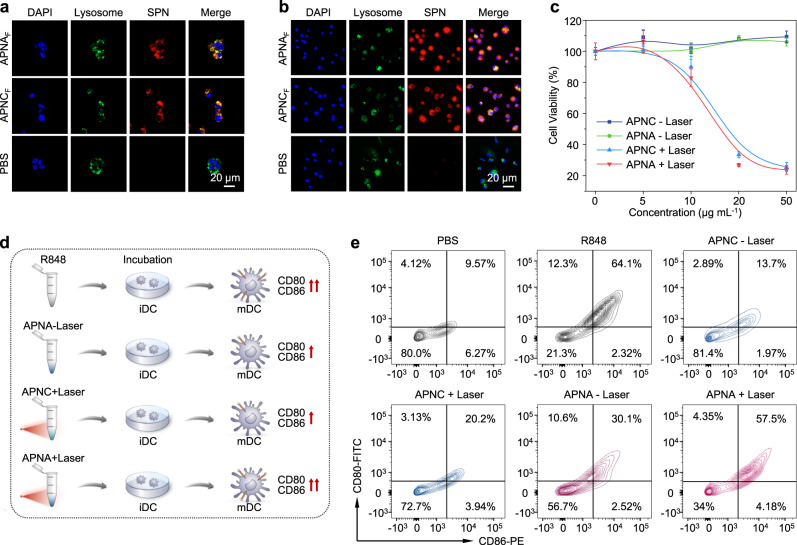
In vitro APNA-mediated photothermal immunotherapy. **a**, **b** Confocal fluorescence images of cellular internalization (24 h) of APNA_F_ and APNC_F_ ([pBODO] = 10 µg mL^−1^) in 4T1 cancer cells **a** and BMDCs **b**. APNA and APNC were labeled with NCBS (2.5 w/w%). Blue fluorescence indicated nuclei staining by 4’, 6-diamidine-2’-phenylindole dihydrochloride (DAPI). Green fluorescence indicated lysosomes staining by LysoTracker Green DND-26. Red fluorescence denoted as SPN channel indicated NIR fluorescence from APNA_F_ or APNC_F_. Experiments were performed in triplicate with similar results. **c** Cell viability assay of 4T1 cells at 12 h after treatment with APNC or APNA at different concentrations with or without 1064 nm photoirradiation (1 W cm^−2^, 6 min) (*n* = 3). **d** Schematic illustration of pre-photothermal activation of APNA and subsequent in vitro stimulation of iDC. **e** Flow cytometry analysis of BMDC maturation (gated on CD11c^+^ DCs) at 48 h after various treatments. [R848] = 4.4 µg mL^−1^; [pBODO] = 10 µg mL^−1^; 1064 nm photoirradiation, 1 W cm^−2^, 6 min. Data were expressed as mean ± SD.

Cellular uptake of APNA and APNC was evaluated on 4T1 murine breast cancer cells and bone marrow-derived DCs (BMDCs). A fluorescent dye (silicon 2,3-naphthalocyanine bis(trihexylsilyloxide)) (NCBS) was doped into nanoparticles (2.5 w/w%) to obtain fluorescent derivatives (APNA_F_ and APNC_F_) (Supplementary Figs. 14, 15). After treating 4T1 cells with APNA_F_ or APNC_F_ for 24 h, strong NIR fluorescence was observed from cellular plasma (Fig. 3a), indicating effective endocytosis of both nanoparticles in 4T1 cells. Similar uptake was detected for BMDCs (Fig. 3b, Supplementary Fig. 16). Furthermore, the nanoparticle signal overlapped well with the green fluorescence from lysosome staining, suggesting that endocytosed nanoparticles mainly resided in lysosomes, wherein TLR7/8 was highly expressed^35,36^. Without photoirradiation, both APNA and APNC induced negligible cytotoxicity to 4T1 cells (Fig. 3c). However, with NIR-II photoirradiation, both nanoparticles triggered significant cell death relative to the control group. For instance, at 50 µg mL^−1^, the cell viability of APNA and APNC treated-group decreased to 24% and 25%, respectively.

The ability of APNA to photothermally trigger in vitro immune activation was investigated on DCs, a crucial species of APCs whose maturation impacts the activation of both innate and adaptive immunity^37^. After photoirradiation of APNA or APNC solution for 10 min, the solutions were transferred to the culture media of immature BMDCs, followed by detection of their maturation by flow cytometry analysis on the expression of the costimulatory molecules CD80 and CD86 (Fig. 3d, Supplementary Fig. 17). The post-photoirradiated APNA triggered upregulation of CD80 and CD86 (57.5%), which was 1.9-fold and 2.8-fold higher than unirradiated APNA (30.1%) or the post-photoirradiated control (APNC) (20.2%) (Fig. 3e). Furthermore, the DC maturation induced by APNA after photothermal activation was similar to free R848 at 4.4 µg mL^−1^. Consistently, mixed lymphocyte reaction indicated the superior T-cell stimulation capacity of pre-activated APNA-treated DCs, as revealed by the higher percentage of CD3^+^CD8^+^ T cells and elevated IFN-γ production relative to other groups (Supplementary Fig. 18). These results show that the immune-stimulating ability of APNA was effectively activated by NIR-II photoirradiation.

### In vivo photothermal immunotherapy

To identify the optimal therapeutic window of APNA and APNC, both nanoparticles were doped with 2.5 w/w% NCBS (APNA_F_ and APNC_F_) (Supplementary Fig. 15), and their accumulation in tumor was monitored by whole-body NIR fluorescence imaging. After systemic administration of APNA_F_ or APNC_F_, NIR fluorescence signals from tumor regions in living mice continuously increased and reached their maxima at 24 h post injection (Supplementary Fig. 19a, b). At this timepoint, fluorescence intensities of APNA_F_ and APNC_F_ were 3.3 and 3.8-fold higher than the background, respectively. Besides, ex vivo biodistribution study at 32 h post injection of nanoparticles indicated that both nanoparticles displayed similar biodistribution (Supplementary Fig. 19c, d). The residual injected nanoparticles had maximum accumulation in tumor, followed by liver, spleen or lung, and other organs. Such preferential tumor accumulation of both nanoparticles suggested their passive tumor-targeting ability owning to the enhanced permeation and retention effect^38^.

**Fig. 4:**
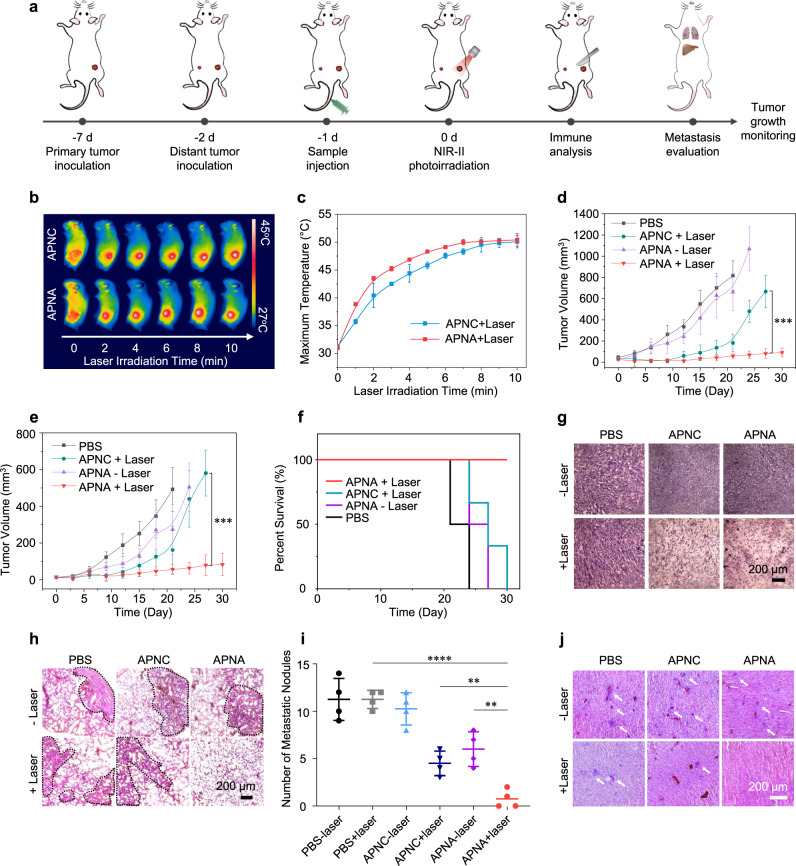
In vivo NIR-II photothermal immunotherapy. **a** Schematic illustration of in vivo anti-cancer therapy. **b** Thermal images of 4T1 tumor-bearing mice during 1064 nm photoirradiation (1 W cm^−2)^ at 24 h post-injection of APNC or APNA (200 µL per mouse, [pBODO] = 250 µg mL^−1^, *n* = 3). **c** Maximum surface tumor temperature of 4T1 tumor-bearing mice during photoirradiation in **b** (*n* = 3). **d**–**e** Tumor growth curves of primary **d** and distant **e** tumor in living mice after various treatments (*n* = 6). APNC + laser and APNA + laser in **d**: *P* = 0.0001; APNC + laser and APNA + laser in **e**: *P* = 0.00029. **f** Survival curves of 4T1-tumor-bearing living mice after various treatments (*n* = 6). **g** H&E staining of tumor in living mice after various treatments in **a**. **h** H&E staining of lung metastatic nodules (indicated by black dotted circle) in 4T1-tumor-bearing mice after various treatments. **i** Number of metastasis nodules per lung in 4T1-tumor-bearing mice after various treatments (*n* = 4). PBS + laser and APNA + laser: *P* < 0.0001; APNC + laser and APNA + laser: *P* = 0.0034; APNA− laser and APNA + laser: *P* = 0.0022. **j** H&E staining of liver metastasis (indicated by white arrows) in 4T1-tumor-bearing mice after various treatments. Data were expressed as mean ± SD. Statistical analysis was performed by two-tailed Student’s *t* test. ns: not significant; *P < 0.05, **P < 0.01; ***P < 0.001; ****P < 0.0001.

Therapeutic efficiency of APNA-mediated NIR-II photothermal immunotherapy was examined on 4T1 tumor-bearing mice. To establish bilateral tumor models, 4T1 cancer cells were subcutaneously inoculated onto the right flank of Balb/c living mice, followed by a second inoculation 5 days later on the left flank of the same mice (Fig. 4a). On the basis of fluorescence imaging results, NIR-II photoirradiation was applied to the primary tumors at 24 h post intravenous administration of APNA or APNC. Upon NIR-II photoirradiation, tumor surface temperatures of both APNA and APNC-treated group continuously increased to 50 °C (Fig. 4b, c).

After therapies, tumor growths curves of both primary and distant tumors were monitored for 30 days. Primary tumors on APNA-injected and photoirradiation-treated mice were nearly completely ablated, and the growth of distant tumors was efficiently inhibited (Fig. 4d, e). However, such high inhibitory efficiencies of primary and distant tumors were not achieved by either APNC-injected and photoirradiation-treated mice or APNA-injected and photoirradiation-treated mice. Furthermore, APNA-injection followed by photoirradiation achieved superior survival benefit than other treatments (Fig. 4f). In addition, no noticeable weight loss was found for mice after all these treatments (Supplementary Fig. 20). The therapeutic efficiency was further confirmed by hematoxylin and eosin (H&E) staining of primary tumors after different therapies (Fig. 4g). Significant nucleus dissociation was clearly observed in primary tumors on APNA or APNC-injected photoirradiation-treated mice after photoirradiation treatments, whereas no obvious cell death was observed in tumors after the other treatments.

Pulmonary metastasis of mice after different therapies was examined by H&E staining (Fig. 4h). Relative to PBS-treated group, fewer metastatic nodules were counted in lungs from APNC-injected and photoirradiation-treated mice or APNA-injected mice without photoirradiation (Fig. 4i). However, almost no metastatic nodules were found on APNA-injected and photoirradiation-treated mice. In line with the results of lung metastasis, no noticeable liver metastatic nodules could be observed on APNA-injected and photoirradiation-treated mice, whereas obvious metastatic nodules were detected on the other groups (Fig. 4j). Further, no obvious histological or pathological changes were found in the other major organs such as hearts, spleens, and kidneys from mice after various therapies, suggesting the good in vivo biocompatibility of both APNA and APNC (Supplementary Fig. 21). Together, these data demonstrated that APNA-mediated NIR-II photothermal immunotherapy not only impeded growth of both primary and distant tumors, but also arrested systemic cancer metastasis to lung and liver, which was not achievable for either monotherapy.

### Activated immune responses after NIR-II photothermal immunotherapy

**Fig. 5:**
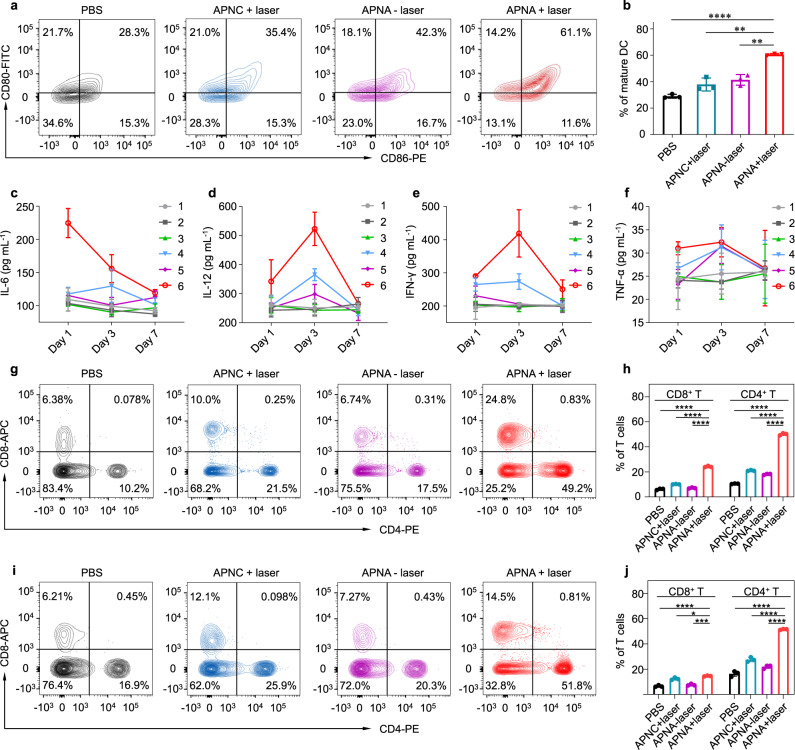
In vivo immune responses after NIR-II photothermal immunotherapy. **a** DC maturation (gated on CD11c^+^ DCs) in tumor-draining lymph nodes from mice after different treatments. **b** Quantification of mature DCs in tumor-draining lymph nodes from mice after different treatments (*n* = 3). PBS + laser and APNA + laser: *P* < 0.0001; APNC + laser and APNA + laser: *P* = 0.0014; APNA− laser and APNA + laser: *P* = 0.0012. **c**–**f** Cytokine levels of IL-6 **c**, IL-12p70 **d**, IFN-ɣ **e**, TNF-α **f** in serum (pg mL^−1^) from mice at day 1, 3, and 7 after different treatments (*n* = 3). 1. PBS injection without photoirradiation; 2. PBS injection with laser irradiation; 3. APNC injection without photoirradiation; 4. APNC injection with photoirradiation; 5. APNA injection without photoirradiation; 6. APNA injection with photoirradiation. **g** Representative flow cytometry plots of CD8^+^ T cells and CD4^+^ T cells in tumor-infiltrating CD45^+^ lymphocytes in primary tumors from mice after various treatments. **h** Quantification of CD8^+^ T cells and CD4^+^ T cells as a percentage of CD45^+^ lymphocytes in primary tumors (*n* = 3). *P* < 0.0001. **i** Representative flow cytometry plots of CD8^+^ T cells and CD4^+^ T cells in tumor-infiltrating CD45^+^ lymphocytes in distant tumors from mice after various treatments. **j** Quantification of CD8^+^ T cells and CD4^+^ T cells as a percentage of tumor-infiltrating CD45^+^ lymphocytes in distant tumors (*n* = 3). *P* values for CD8^+^ T cells: PBS + laser and APNA + laser: *P* < 0.0001; APNC + laser and APNA + laser: *P* = 0.021; APNA − laser and APNA + laser: *P* = 0.0001. *P* values for CD4^+^ T cells: *P* < 0.0001. Data were expressed as mean ± SD. Statistical analysis was performed by two-tailed Student’s *t* test. ns: not significant; *P < 0.05, **P < 0.01; ***P < 0.001; ****P < 0.0001.

To investigate the immune response after various therapies, activation of DCs in tumor-draining lymph nodes was first evaluated by flow cytometry. Consistent with in vitro results (Fig. 3e), APNA-mediated photothermal immunotherapy triggered the highest level of DC maturation (61%) among all treatments (Fig. 5a, b), which was 1.7-, 1.5-, and 2.2-fold higher than APNC injection together with photoirradiation treatment (APNC-mediated PTT) (35%), monotreatment of APNA (42%) and the control group (28%), respectively. In addition, proinflammatory cytokines including interleukin 6 (IL-6), interleukin 12 (IL-12p70), interferon ɣ (IFN-ɣ), and tumor necrosis factor α (TNF-α) in sera of mice were analyzed by enzyme-linked immunosorbent assay (ELISA) over time after various treatments (Fig. 5c–f). In line with results of DC maturation, APNA-mediated photothermal immunotherapy aroused the highest levels of four kinds of cytokines among all treatments at each timepoint. For instance, at day 1 after treatment, the IL-6 level aroused by APNA-mediated photothermal immunotherapy (224 pg mL^−1^) was 1.9-fold higher than APNC-mediated PTT (117 pg mL^−1^) or monotreatment of APNA (115 pg mL^−1^).

Cytotoxic T lymphocytes (CTL) (CD8^+^ T cells) play essential roles in directly combating cancer cells and helper T cells (CD4^+^ T cells) are critical to regulating adaptive immunities^9^. Therefore, immune cells in both primary and distant tumors from mice after various treatments were analyzed by flow cytometry. APNA-mediated photothermal immunotherapy caused the highest CD8^+^ T-cell infiltration into both primary (24.8%) and distant tumors (14.5%) (Fig. 5g–j). In detail, the efficiency of CTL recruitment after APNA-mediated photothermal immunotherapy was 2.5- and 3.7-fold higher than APNC-mediated PTT or APNA alone in primary tumor, and 1.2- and 2.0-fold higher in distant tumor, respectively. In addition to CTL recruitment, APNA-mediated photothermal immunotherapy triggered the most significant infiltration of helper T cells in both primary and distant tumors (Fig. 5g–j), showing 2.3- or 2.8-fold increase in population than APNC-mediated PTT or APNA without photoirradiation. Consistently, the populations of both CD8^+^ cytotoxic T cells and CD4^+^ T helper cells in the spleen and blood were most significantly elevated after APNA-mediated photothermal immunotherapy (Supplementary Figs. 22, 23). Further, flow cytometry analysis suggested the highest generation of tumor-specific CTLs in living mice after APNA-mediated photothermal immunotherapy rather than other treatments (Supplementary Fig. 24). Moreover, the superior antitumor efficacy of ANPA-mediated photothermal immunotherapy was not observed in tumor models on T-cell-deficient NCr nude mice in comparison with APNC-mediated PTT (Supplementary Fig. 25), validating the essential role of T-cell-mediated immune response to efficiently inhibit tumor growth in NIR-II photothermal immunotherapy. These data validated that APNA-mediated photothermal immunotherapy stimulated superior systemic immune response over other monotreatment, which was the origin of efficient inhibition of cancer metastasis (Fig. 4e, h–j).

### Mechanistic study of photothermal immunoactivation in deep tumor

**Fig. 6:**
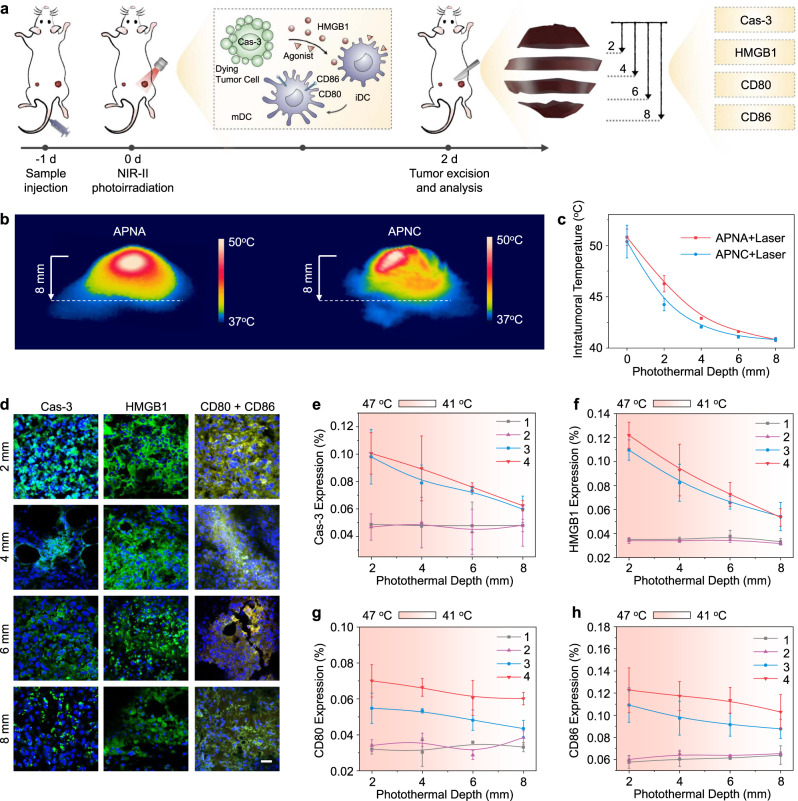
Mechanistic study of photothermal activation and immunogenicity in deep tumor. **a** Scheme of photothermal depth of tumor after various treatments in Fig. 4 and study of immunogenic response at each photothermal depths. **b** Thermal images of tumor in the direction of photothermal depth. **c** Quantification of intratumoral temperature in Fig. 6b as a function of photothermal depth (*n* = 3). **d** Immunofluorescent images of Cas-3, HMGB1, and CD80/CD86 in tumor sections at different photothermal depths after APNA-mediated photothermal immunotherapy. Cas-3, HMGB1, and CD80 were indicated by green pseudo colors, whereas CD86 was indicated by orange pseudo color. Blue fluorescence indicated nuclei staining by DAPI. Scale bar: 20 µm. **e**–**h** Quantification of expression levels of Cas-3 **e**, HMGB1 (in cytoplasm) **f**, CD80 **g**, CD86 **h** in tumors after various treatments as a function of photothermal depth. Treatments: 1. PBS + laser; 2. APNA − laser; 3. APNC + laser; 4. APNA + laser. Data were expressed as mean ± SD. Error bars indicated standard deviations of three independent measurements.

The underlying mechanism of superior immune activation effect by APNA-mediated photothermal immunotherapy was investigated at molecular level. Although immunogenicity has been well demonstrated in PTT^39^, the detailed activation process in deep tumor remains ambiguous owing to the heterogenous heat distribution in solid tumor. In this regard, immune activation by APNA-mediated photothermal immunotherapy was studied at different tumor depths in the direction of photoirradiation (termed as photothermal depth) and compared with APNC-mediated PTT (Fig. 6a). At 10 min during photoirradiation, intratumoral temperatures at various tissue depths were monitored and analyzed (Fig. 6b). As expected, both APNC-mediated PTT and APNA-mediated photothermal immunotherapy induced gradually decreased intratumoral temperature with the increase of photothermal depth (Fig. 6c). After various treatments, primary tumors from living mice were harvested and dissected at different photothermal depths. At each depth, expression levels of representative biomarkers of cellular apoptosis (caspase-3, termed as Cas-3), ICD (high mobility group box 1 protein, termed as HMGB1), and DC maturation (CD80 and CD86) were examined by immunofluorescent staining of tumor sections (Fig. 6d, Supplementary Figs. 26–28).

Quantitative analysis indicated that both APNC-mediated PTT and APNA-mediated photothermal immunotherapy aroused higher expression of biomarkers than APNA or PBS treatment at each photothermal depth (Fig. 6e–h). With the increase of photothermal depth, the expression levels of four biomarkers were downregulated in both photothermal immunotherapy and PTT, whereas almost no obvious fluctuation in biomarkers was observed in APNA or PBS treatment. Intriguingly, no significant discrimination in cellular apoptosis or ICD was observed between PTT and photothermal immunotherapy, whereas obvious upregulation in expression of costimulatory factors CD80 and CD86 was found in photothermal immunotherapy over PTT at each photothermal depth.

Further, relationship between immunogenicity and intratumoral temperature was analyzed (Fig. 6e–h). Consistent with previous reports^40^, the extent of cellular apoptosis, ICD triggered by hyperthermia and maturation of DC displayed temperature-dependent pattern in tumor tissue. Specifically, at similar intratumoral temperature, both PTT and photothermal immunotherapy triggered nearly identical degree of cellular apoptosis or ICD. However, the degree of DC maturation in photothermal immunotherapy was significantly elevated in comparison with PTT at the same tumor temperature. For instance, at ~44 °C, the expression level of CD80 in photothermal immunotherapy was 20% higher than that by PTT. Given the temperature-dependent liberation of pro-agonist (Fig. 2i), the higher DC maturation in photothermal immunotherapy than PTT should be assigned to the activated agonist from APNA. These data thus not only disclosed the gradient distribution of immunogenicity along the photothermal depth in primary tumor, but also highlighted the potentiation of immune response by precise photothermal activation of caged TLR agonist.

## Discussion

Photothermal activation reliant on heat produced by molecular transducers upon photoirradiation provides non-invasive way to remote control of biological or therapeutic process^41–43^. In addition to precise regulation, photothermal heat is able to locally ablate solid tumors and induce ICD, ultimately contributing to enhanced antitumor immunity^39^. Despite the growing interest of photothermal activation in nanomedicine, its integration with immunotherapy has been scarcely reported. We herein synthesized the first polymer nanoagonist (APNA) that could be specifically activated by NIR-II photothermia to trigger the liberation of covalently conjugated immunostimulants. Such a molecular regulation by external light confined the region of immune activation, potentially minimizing the off-target immune response towards normal organs. Owing to the superior tissue penetration depth of NIR-II over NIR-I light, APNA allowed for efficient tumor ablation and potentiated immunogenicity at up to 8 mm in deep solid tumor, which was not reachable for NIR-I photoimmunotherapy^40^.

Development of multifunctional photothermal nanotransducers with strong absorption above 1000 nm is imperative for combinational NIR-II phototherapy. However, most existing NIR-II photothermal agents are derived from inorganic materials, which encounter the potential issue of leaking heavy-metal ions to cause chronic toxicity. Differently, SPNs are intrinsically made of organic polymers with good biocompatibility. Through rational donor-acceptor engineering, the semiconducting backbone of APNA was designed to have excellent PCE of 84.4% in the NIR-II region, which outperformed the majority of the NIR-II photothermal agents (e.g., gold nanostructures^25^, copper-derived nanoparticles^44^, 2D Nb_2_C nanosheets^45^, etc.) reported so far. In addition, the chemical flexibility of SP allowed for potent conjugation of polymer precursor pBODO with pro-therapeutics through thermosensitive linker. Thus, APNA favored both deep-tissue photothermal ablation and in vivo molecular activation. Despite that R848 has been investigated as a potent topically administered TLR agonist in preclinical settings, its application in systemic therapy is hampered due to the dose-limiting adverse effects such as transient peripheral blood leukocyte depletion^46–48^. Thus, R848 has been physically encapsulated into nanoparticle through hydrophobic interactions, which however potentially has the issue of nonspecific dissociation and leakage during blood circulation. In contrast, our activatable design circumvented these issues, because R848 was molecularly masked and covalently conjugated to the side chain of APNA via a thermolabile linker. Only upon NIR-II photoirradiation, R848 could be uncaged from APNA and became molecularly active, permitting in situ intertumoral immunomodulation with high spatiotemporal resolution. Such precise regulation in turn allowed for a much lower (2.4–24 folds) injected dosage of TLR agonist (0.25 mg kg^−1^) than those used in previous studies (0.6–6 mg kg^−1^)^7,9,10,49,50^, further reducing the risk of irAEs.

Immunostimulatory activities aroused during photothermal treatment have been studied, whereas the intratumoral details remain unclear. By virtue of the deep-tissue photothermal therapeutic capability of ANPA, intratumoral immune activation process was studied and its relationship with photothermal depth as well as intratumoral temperature was unveiled. In both PTT and photothermal immunotherapy, along the direction of photoirradiation, it was observed that intratumoral photothermal temperature gradually decreased, presumably leading to attenuated cellular apoptosis, extent of ICD, and DC maturation. More importantly, despite the varied intratumoral temperatures, the degree of elevated DC maturation assigned to photothermal activation of ANPA remained nearly identical at different photothermal depths (Fig. 6g, h). This should be the low decomposition temperature of thermolabile linker (~44 °C) and favorable permeation of released small agonist in tumor interstitium. Such deep-tumor immune stimulation by APNA-mediated photothermal immunotherapy potentiated antitumor systemic immunity, contributing to elevated CTL and helper T-cell infiltration in not only residual primary tumor but also distal tumor and spleen to inhibit cancer metastasis, which was not possible for NIR-II PTT alone.

In summary, we developed NIR-II photothermally activatable polymeric pro-nanoagonist for combinational photothermal immunotherapy of cancer. This work not only illustrates a remote approach to spatiotemporal control of immune activation to reinforce antitumor effect but also unveils the intratumoral mechanism of immunogenicity during photothermal activation and treatment. In addition to TLR agonists, this strategy could be generalized for the development of other light-responsive immunotherapeutic nanoagents simply by conjugating other small-molecule immunostimulants or checkpoint inhibitors to the polymer.

## Methods

### Materials

All chemicals were purchase from Sigma-Aldrich unless otherwise indicated. 4,8-Bis[5-bromo-4-(2-octyldodecyl)-2-thienyl]-benzo[1,2c:4,5c′]bis[1,2,5]thiadiazole (BBT), 2,5-bis(6-bromohexyl)-3,6-bis(5-bromothiophen-2-yl)pyrrolo[3,4-c]pyrrole-1,4(2H,5H)-dione (DPP-Br) and (4,8-bis((2-ethylhexyl)oxy)benzo[1,2b:4,5-b′]dithiophene-2,6-diyl)bis(trimethylstannane) (OT) were purchased from Luminescence Technology Corp (Lumtec). Mouse granulocyte macrophage-colony stimulating factor (GM-CSF) was purchased from i-DNA Biotechnology Pte Ltd. HMGB1 antibody (Catalog no. 3935 S, dilution: 1:100) and cleaved caspase-3 antibody (Catalog no. 9661 L, dilution: 1:500) were purchased from Cell Signaling Technology. Mouse ELISA kits for TNF-α, IL-6, IL-12(p70), IFN-ɣ were purchased from Biolegend. APC anti-mouse CD11c (Catalog no. 117310, dilution: 1:80), FITC anti-mouse CD80 (Catalog no. 104706, dilution: 1:50), PE anti-mouse CD86 (Catalog no. 105008, dilution: 1:20), FITC anti-mouse CD3 (Catalog no. 100204, dilution: 1:50), APC anti-mouse CD8 (Catalog no. 100712, dilution: 1:80), purified anti-mouse CD16/32 (Catalog no. 156604, dilution: 1:200) and Alexa Fluor® 700 anti-mouse CD45 (Catalog no. 103128, dilution: 1:200), PE anti-mouse CD4 (Catalog no. 130310, dilution: 1:80) were purchased from Biolegend. Live/Dead^TM^ Fixable Blue Dead Cell Stain was purchased from Thermo Fisher Scientific (Catalog no. L23105, dilution: 1:1000). Secondary antibody Alexa Fluor 488 conjugated goat anti-rabbit IgG was purchased from Thermo Fisher Scientific (Catalog no. 2045215, dilution: 1:500). R848 was purchased from MedKoo Biosciences. VA-044 was purchased from Scientific Hub Services Pte Ltd. HO-PEG2000-COOH was purchased from JenKem Technology USA Inc.

### Instrumentation

Absorption spectra of nanoparticles were measured by Lambda 950 spectrometer. Fluorescence spectra of nanoparticles were measured by Fluorolog 3-TCSPC spectrometer. 1H NMR of intermediates was measured on Bruker Avance II 300 MHz NMR spectrometer. DLS and zeta potential profiles were measured on Malvern Nano-ZS Particle Sizer. TEM images were captured by JEOL JEM 1400 transmission microscope (accelerating voltage 100 kV). Photothermal temperature of solution and tumor was measured by FLIR thermal camera (T420). Laser source (1064 nm) was purchased from Shanghai Connet Fiber Optics. HPLC was performed on an Agilent 1260 system with ACN (0.1% of trifluoroacetic acid) and H_2_O (0.1% of trifluoroacetic acid) as the eluent. LCMS spectra were measured on Thermo Finnigan Polaris Q quadrupole ion trap mass spectrometer equipped with a standard electrospray ionization (ESI) source. Flow cytometry was measured on Fortessa X20 (BD Biosciences). Confocal fluorescence images were captured on Zeiss LSM 800 confocal laser scanning microscope. In vivo NIR fluorescence imaging was performed on IVIS imaging system (PerkinElmer). Tumor and organ tissues were dissected on a cryostat (Leica). H&E images of tissue sections were captured on ECLIPSE 80i microscope (Nikon). NMR spectra were analyzed using Mestre Nova LITE v5.2.5-4119 software (Mestre lab Research S.L.).

### Synthesis of pBODO-N_3_

BBT (29 mg, 0.027 mmol), DPP-Br (21 mg, 0.027 mmol), OT (42 mg, 0.054 mmol), Pd_2_(dba)_3_ (0.6 mg, 0.0006 mmol), and tri(o-tolyl)phosphine (1.8 mg, 0.005 mmol) were weighed and added to round-bottom flask (50 mL), followed by addition of chlorobenzene (8 mL). The mixture was degassed through three freeze–thaw cycles and the reaction was then performed at 100 °C under N_2_ atmosphere for 2 h. After reaction, the resulted solution was precipitated in cold methanol. Then, precipitates were collected through centrifugation, washed three times with methanol and dried under vacuum to obtain pBODO-Br powder (95% yield). Thereafter, pBODO-Br (16 mg) was dissolved in a mixture of THF and DMF (THF: DMF 2:1) followed by addition of sodium azide (16 mg) and stirring at room temperature for 2 days. Then, the obtained solution was concentrated by rotary evaporation, dissolved in dichloromethane, washed three times with water and then dried over anhydrous sodium sulfate. Thereafter, the solvent was removed by rotary evaporation and the obtained solid was dried under vacuum to afford pBODO-N_3_ (98% yield). ^1^H NMR (300 MHz, CDCl_3_): δ 7.29–7.41 (m, 2H), 6.98 (s, 8H), 5.36–5.01 (m, 4H), 3.72–3.18 (m, 4H), 2.82–2.35 (m, 4H), 2.27 (m, 8H), 1.43 (m, 66H), 1.25 (m, 68H), 0.85 (m, 24H).

### Synthesis of VR

VA-044 (130 mg, 0.4 mmol) was dissolved in dry ACN, followed by addition of NaH (16 mg, 0.67 mmol) and stirring at room temperature for 30 min. Thereafter, 7-iodoheptanoic acid (236 mg, 0.92 mmol) was dissolved in dry ACN and added to the reaction mixture. After stirring at room temperature for 2 days, the solvent was removed by rotary evaporation and the obtained solid was purified with HPLC to afford pure product VCOOH (22% yield). ^1^H NMR (300 MHz, CD_3_OD): δ 3.7–3.4 (m, 12H), 1.93 (m, 4H), 1.61 (s, 12H), 1.51 (m, 4H), 1.34 (m, 12H). ESI-MS (m/z): [M]^+^ calcd. for VCOOH, 506.36; found, 506.62.

VCOOH (45 mg, 0.09 mmol), R848 (14 mg, 0.045 mmol), EDC (8.64 mg, 0.045 mmol), and DMAP (5.5 mg, 0.045 mmol) were weighed and added to round-bottom flask, followed by addition of dry ACN (9 mL) and stirring at room temperature for 3 days. After reaction, the solvent was removed by rotary evaporation and the residues were purified by HPLC to obtain pure product VR (34% yield). ^1^H NMR (300 MHz, CD_3_OD): δ 8.26 (d, 1H), 7.54 (d, 1H), 7.45 (m, 1H), 7.15 (m, 1H), 5.0 (s, 2H), 4.12 (s, 2H), 3.53 (m, 2H), 3.36 (m, 12H), 2.18-1.98 (m, 8H), 1.98–1.80 (s, 12H), 1.26 (m, 12H), 1.15–1.05 (s, 9H). ESI-MS (m/z): [M-H]^+^ calcd. for VR, 802.52; found, 800.79.

### Synthesis of alkyne-PEG-VR

HO-PEG-COOH (500 mg, 0.25 mmol) and NaH (100 mg, 4.2 mmol) were weighed, dissolved in dry THF and stirred on ice bath for 1 h. Then, NaH (50 mg, 2.1 mmol) and propargyl bromide (30 µL, 0.38 mmol) were added to the reaction mixture and stirred overnight. After reaction, the reaction solution was filtered to remove large precipitates. Thereafter, the crude product was concentrated via rotary evaporation and precipitated in cold diethyl ether. The precipitates were collected via centrifugation, followed by dialysis and lyophilization to obtain powder of carboxyl-PEG-alkyne. Carboxyl-PEG-alkyne (100 mg, 0.05 mmol), EDC (96 mg, 0.5 mmol), and NHS (57 mg, 0.5 mmol) were weighed and dissolved in anhydrous THF, and then stirred at room temperature for 30 min. Then ethylenediamine (34 µL, 0.5 mmol) was dropwise added to the reaction mixture followed by stirring for 2 days. After reaction, THF was removed by rotary evaporation and the residues were purified by dialysis and lyophilization to obtain pure alkyne-PEG-NH_2_ (80% yield). ^1^H NMR (300 MHz, CDCl_3_): δ 4.21 (s, 2H), 3.99 (s, 2H), 3.88 (m, 2H), 3.65 (m, 176H), 2.45 (m, 3H).

VR (16 mg, 0.02 mg), EDC (24 mg, 0.12 mmol), and NHS (14 mg, 0.12 mmol) were weighed and stirred in anhydrous THF at room temperature for 30 min. Then alkyne-PEG-NH_2_ (24 mg, 0.012 mmol) dissolved in dry THF was dropwise added to the reaction mixture and stirred together for 2 days. After reaction, the solvent THF was removed by rotary evaporation and the residues were purified by dialysis and lyophilization to obtain pure alkyne-PEG-VR. ^1^H NMR (300 MHz, CDCl_3_): δ 7.27–7.52 (m, 3H), 7.11 (d, 1H), 4.21–3.88 (m, 4H), 3.65 (s, 152H), 3.41 (m, 4H), 2.84 (s, 1H), 2.22 (m, 4H), 1.56 (m, 13H), 1.26 (m, 28H).

### Synthesis of pBODO-PEG and pBODO-PEG-VR

pBODO-N_3_ (5 mg), alkyne-PEG-VR (17 mg), CuBr (5 mg), and PMDETA (35 µL) were weighed and dissolved in anhydrous THF. Then the reaction was carried out under N_2_ atmosphere at room temperature for 2 days. After reaction, THF was removed by rotary evaporation and the residues were dissolved in deionized water followed by dialysis and lyophilization to obtain pBODO-PEG-VR (90% yield). pBODO-PEG was synthesized by repeating the same click procedure except for substituting methoxy-PEG-alkyne for alkyne-PEG-VR.

### Preparation of APNA and APNC

To prepare APNA, pBODO-PEG-VR powder was dissolved in THF ([pBODO] = 1 mg mL^−1^) and then rapidly injected into water under sonication, followed by evaporation of THF under a gentle N_2_ flow to enable self-assembly into APNA. The obtained solution was then filtered through Millipore 220 nm polyvinylidene fluoride filter to remove large impurities. The filtered solution was then concentrated by ultracentrifugation at 3260 × *g* for 25 min at 4 °C to obtain APNA stock solution. APNC was prepared by repeating the same step, whereas substituting pBODO-PEG-VR with pBODO-PEG.

### In vitro photothermal study

For photostability test, APNA or APNC solution (([pBODO] = 20 µg mL^−1^) was irradiated with 1064 nm laser (1 W cm^−2^) for 6 min, followed by a natural cooling process for another 6 min. Such heating-cooling cycles were repeated five times and solution temperature was monitored by IR thermal camera. Photothermal conversion efficiency of APNC was measured according to previous report^25^. In brief, APNC (optical density at 1064 nm = 1, 2 mL) was placed in a quartz cuvette (5.66 g, 3.5 mL, Sangon Biotech, Shanghai, China). Then the solution was irradiated with 1064 nm laser (1 W cm^−2^) for 30 min followed by removal of laser and a subsequent natural cooling process for another 30 min. Solution temperature during laser irradiation and natural cooling was monitored by a dual input J/K type thermometer (TM300, Extech Instruments). Thereafter, photothermal conversion efficiency was calculated according to the above-mentioned report as shown below^25^.

In brief, the total energy input and dissipation of the above-mentioned system could be interpreted as:

1 $$\mathop {\sum }\limits_i m_iC_{p,i}\frac{{dT}}{{dt}} = Q_{NP} + Q_{sys} - Q_{diss}$$

where *m*_*i*_ and *C*_*p,i*_ are, respectively, the mass and heat capacity of the constituents such as water and quartz cuvette. *Q*_*NP*_ is the energy input by photoirradiated APNC; *Q*_*sys*_ is the energy input contributed by the other constituents; and *Q*_*diss*_ is the energy loss from measurement system to the ambient environment. *Q*_*NP*_ could be expressed as:

2 $$Q_{NP} = I\left( {1 - 10^{ - {\mathrm{A}}_\lambda }} \right)\eta$$

where *I* is laser power; *A*_*λ*_ is the optical density of APNC at 1064 nm; *η* is photothermal conversion efficiency. Besides, *Q*_*diss*_ could be interpreted as:

3 $$Q_{diss} = hS\left( {T - T_{surr}} \right)$$

Where *h* stands for heat transfer coefficient; *S* is surface area that is exposed to photoirradiation; *T*_*surr*_ is the temperature of surroundings. When the thermal equilibrium of the measurement system is reached, temperature is recorded as *T*_*max*_. At this point in time, total energy input equals to the energy dissipation:

4 $$Q_{NP} + Q_{sys} = Q_{diss} = hS\left( {T_{{\it{max}}} - T_{surr}} \right)$$

After stoppage of photoirradiation, the energy input equals zero. Therefore Eq. (1) is interpreted as:

5 $$\mathop {\sum }\limits_i m_iC_{p,i}\frac{{dT}}{{dt}} = - Q_{diss} = - hS\left( {T - T_{surr}} \right)$$

After rearrangement and integration, Eq. (5) is expressed as:

6 $$t = - \frac{{\mathop {\sum }\nolimits_i m_iC_{p,i}}}{{hS}}{\mathrm{ln}}\frac{{T - T_{surr}}}{{T_{{\it{max}}} - T_{surr}}}$$

The time constant *τ*_*s*_ stands for:

7 $$\tau _s = \frac{{\mathop {\sum }\nolimits_i m_iC_{p,i}}}{{hS}}$$

To simplify Eq. (6), a dimensionless factor *θ* is defined:

8 $$\theta = \frac{{T - T_{surr}}}{{T_{{\it{max}}} - T_{surr}}}$$

Therefore Eq. (6) is presented as:

9 $$t = - \tau _s{\mathrm{ln}}\theta$$

Thereafter, *τ*_*s*_ is calculated through linear correlation of time and *lnθ*. And *hS* is calculated by Eq. (7). On the other hand, *Q*_*sys*_ is measured by altering APNC solution to the solvent:

10 $$Q_{sys} = hS\left( {T_{{\it{max}},H_2O} - T_{surr}} \right)$$

Ultimately, *η* is calculated as:

11 $$\eta = \frac{{hS\left( {T_{{\it{max}}} - T_{surr}} \right) - Q_{sys}}}{{I\left( {1 - 10^{ - {\mathrm{A}}_\lambda }} \right)}}$$

### In vitro photothermal activation of APNA

To study the photothermal liberation of agonist, APNA solution (20 µg mL^−1^, 200 µL) was irradiated with 1064 nm laser (1 W cm^−2^) for 10 min. Then APNA solutions with or without photoirradiation were filtered through 220 nm filter and analyzed by HPLC using 1% ACN (0.1% trifluoroacetic acid) as the eluent and detection wavelength was 280 nm. Free R848 was analyzed as a control by HPLC using the same method. The product from photothermal cleavage of APNA was purified by HPLC, followed by lyophilization and characterization by ^1^H NMR and ESI-MS. ^1^H NMR (300 MHz, CD_3_OD): δ 8.0 (d, 1H), 7.68 (d, 1H), 7.28 (m, 2H), 5.06 (s, 2H), 4.08 (s, 2H), 3.8-3.4 (m, 8H), 2.22 (m, 2H), 1.47–1.37 (m, 14H), 1.35–1.20 (s, 9H). ESI-MS (m/z): [M+H]^+^ calcd. for this product, 552.34; found, 553.24.

To investigate the relationship of photothermal temperature and photothermal activation ratio, APNA solutions (50 µg mL^−1^, 200 µL) were irradiated with 1064 nm laser with various power densities to keep solution temperature at 37 °C, 45 °C or 55 °C for 20 min. Every 5 min during photoirradiation, products of photothermal activation were collected and analyzed by HPLC using the abovementioned method. To quantify the total amount of drug release, the APNA solution (50 µg mL^−1^, 200 µL) was incubated at 55 °C for 24 h and the products were analyzed using HPLC.

After photoirradiation, APNA solutions were subject to incubation with esterase (1 U) at 37 °C overnight. The hydrolyzed final product was characterized by HPLC (using the aforementioned method) and LCMS. ESI-MS (m/z): [M+Na]^+^ and [M+K]^+^ calcd. for hydrolyzed final product, 314.17; found, 338.40 and 353.01.

### Preparation of APNA_F_ and APNC_F_

pBODO-PEG-VR and 2.5 w/w% NCBS were weighed and dissolved in THF ([pBODO] = 1 mg mL^−1^, [NCBS] = 25 µg mL^−1^). Then APNA_F_ was prepared by repeating the same step as preparation of APNA. Similarly, APNC_F_ was prepared by changing pBODO-PEG-VR into pBODO-PEG.

### In vitro cellular uptake

4T1 murine mammary carcinoma cells were purchased from American Type Culture Collection (ATCC) and cultured in Dulbecco’s modified Eagle medium supplemented with 10% fetal bovine serum and 1% antibiotics (penicillin and streptomycin) at 37 °C in an incubator providing a humid atmosphere containing 5% CO_2_. BMDCs were isolated from bone marrow of Balb/c mice according to established protocols^51^. In brief, femur and tibia were collected from healthy Balb/c mice, and both ends of the bones were carefully trimmed to expose the bone marrow. A syringe needle was inserted to the bones and cells were flushed out by slowly injecting ice-cold RPMI 1640 complete culture medium. The collected contents were filtered through 100 µm cell strainer (Falcon®) to 50-mL centrifuge tube. After centrifugation (850 × *g* for 5 min) and red cell lysis, the remaining cells were resuspended in RPMI 1640 complete culture medium supplemented with 20 ng mL^−1^ GM-CSF. The culture was replenished with fresh culture medium on day 3. At day 6, the BMDCs were harvested by collecting the non-adherent and loosely adherent cells from the suspension. The isolated BMDCs were cultured in RPMI 1640 complete culture medium supplemented with 20 ng mL^−1^ GM-CSF. To investigate cellular uptake of nanoparticles, 4T1 cells or BMDCs were seeded in confocal dishes with a population of 1 × 10^4^ cells per dish and cultured in incubator overnight. Then, APNA_F_ or APNC_F_ ([pBODO] = 10 µg mL^−1^) was added to the dish and cultured with cells for 24 h. Thereafter, the supernatant in the dish was carefully removed and cells were gently washed with fresh PBS, followed by staining with LysoTracker Green DND-26 (Thermo Fisher Scientific) and 4′,6-diamidino-2-phenylindole (DAPI). Then cells were imaged under confocal microscope.

### In vitro anti-cancer therapy

4T1 cells were seeded in 96-well plates (1 × 10^4^ cells per well) and cultured in the incubator overnight. Then APNA or APNC nanoparticles were added to the plates with different concentrations (0, 5, 10, 20, 50 µg mL^−1^) and incubated with cells for 24 h. After incubation, cells were treated with or without 1064 nm laser irradiation (1 W cm^−2^, 6 min). Then cells were incubated for another 24 h and gently washed with PBS. Thereafter, freshly prepared MTS (Promega) working solution (100 µL DMEM plus 20 µL MTS reagent) was added to each well and incubated with cells for 3 h. After reaction, the plate was measured on SpectraMax M5 microplate reader to read the absorbance at 490 nm. Cell viabilities were thus calculated as the ratio of the absorbance of cells with various treatments to that of the control cells without any treatments.

### In vitro photothermal activation of APNA

Prior to incubation with cells, APNA and APNC nanoparticles were irradiated with 1064 nm laser (1 W cm^−2^) for 6 min. BMDCs were seeded in six-well plates and cultured overnight. Then, free R848 (4.4 µg mL^−1^), APNC nanoparticles with or without laser pre-treatment ([pBODO] = 10 µg mL^−1^), APNA nanoparticles with or without laser pre-treatment ([pBODO] = 10 µg mL^−1^) were added to wells and incubated with cells for 48 h, respectively. After incubation, cells were trypsinized, collected, and gently washed with fresh PBS. Thereafter, cells were stained with APC anti-mouse CD11c, FITC anti-mouse CD80 and PE anti-mouse CD86, followed by flow cytometry analysis. Mature DCs were gated as CD11c^+^CD80^+^CD86^+^ cells.

### In vitro T-cell stimulation capacity of DCs

Stimulated DCs obtained in the previous paragraph were co-cultured with CD8^+^ T cells at a DC:T ratio of 10:1 in 24-well plate (10^5^ CD8^+^ T cells per well). T cells were isolated from spleens of healthy Balb/c mice via Dynabeads® Untouched™ Mouse CD8 Cells kit. Mixed lymphocytes were cultured for 3 days. Thereafter, the IFN-γ in the supernatant of culture medium was measured by ELISA kits according to manufacturer’s protocol. And proliferation of CD3^+^CD8^+^ T cells were analyzed by flow cytometry.

### In vivo fluorescence imaging

Animal experiments were performed in compliance with Guidelines for Care and Use of Laboratory Animals of the Nanyang Technological University-Institutional Animal Care and Use Committee (NTU-IACUC) and approved by the Institutional Animal Care and Use Committee (IACUC) for Animal Experiment, Singapore. Mice used in this manuscript were purchased from InVivos Pte Ltd (Singapore). Mice were housed in a temperature controlled (22 °C) room with 12 h dark light cycles (0700 h on and 1900 h off) and 40–70% humidity. 4T1 cells (2 million suspended in 100 µL DMEM) was subcutaneously injected into the right flank of female Balb/c mice (BALB/cNTac, 5 weeks old). In vivo fluorescence imaging was conducted on living mice at 7 days after tumor inoculation. In brief, mice were anesthetized by isofluorene and intravenously injected with APNA_F_ or APNC_F_ (200 µL per mouse, [pBODO] = 250 µg mL^−1^, *n* = 3) and NIR fluorescence from mice was long-termly monitored by IVIS fluorescence imaging system with excitation wavelength at 710 nm and emission wavelength at 780 nm.

At 32 h post injection of nanoparticles, mice were euthanized and both tumors and major organs (hearts, liver, spleens, lungs, and kidneys) were harvested. NIR fluorescence from tumors and organs were measured on IVIS fluorescence imaging system with excitation wavelength at 710 nm and emission wavelength at 780 nm and analyzed using Living Image software.

### In vivo photothermal immunotherapy on Balb/c mice

To inoculate the primary tumor, 4T1 cells (2 million suspended in 100 µL DMEM) was subcutaneously injected into the right flank of female Balb/c mice (BALB/cNTac, 5 weeks old). Five days later, 4T1 cells (0.5 million cells suspended in 50 µL DMEM) was subcutaneously injected into the left flank of Balb/c mice for secondary tumor inoculation. At 1 day post tumor inoculation, mice were intravenously injected with PBS, APNA, or APNC nanoparticles (200 µL per mouse, [pBODO] = 250 µg mL^−1^, *n* = 6). At 24 h post injection, tumors of mice for PTT or photothermal immunotherapy were irradiated with 1064 nm laser at 1 W cm^−2^ for 10 min. Tumor temperatures were monitored by IR thermal camera. Thereafter, tumor volumes of both primary and distant tumors and body weights of measured every 2 days for 30 days. And tumor volumes were calculated according to below formula:

$${\mathit{V}} = \left( {{\mathrm{tumor}}\,{\mathrm{length}}} \right) \times \left( {{\mathrm{tumor}}\,{\mathrm{width}}} \right)^2 \times 0.5$$

And relative tumor volume was expressed as *V*/*V*_0_, where *V*_0_ stood for the initial tumor volume before treatment.

### Histological studies

At day 2 after various therapies, mice were euthanized, and tumors were harvested and fixed in 4% paraformaldehyde (PFA). Then, the fixed tumors were embedded in paraffin and sectioned into 10-µm slices according to standard protocol. To examined cellular necrosis, the obtained tumor sections were stained with H&E and examined under Nikon ECLIPSE 80i microscope.

### Examination of lung and liver metastasis

Mice were killed, and major organs including lungs, livers, spleens, kidneys, and hearts were harvested and fixed in 4% PFA. Numbers of lung metastatic nodules were carefully counted. Thereafter, the fixed organs were embedded in paraffin, dissected to 10-µm slices, and stained with H&E according to standard protocols. Tissue sections of these organs were then examined under Nikon ECLIPSE 80i microscope.

### Cytokine detection

At day 1, 3, and 7 after various therapies, blood was sampled from living mice in heparinized capillary tubes and serum was isolated by centrifugation for analysis. Levels of IL-6, IL-12, IFN-ɣ, and TNF-α were measured by ELISA kits according to standard protocols.

### Ex vivo analysis of immune response

To investigate in vivo DC maturation, mice after various therapies were killed and tumor-draining lymph nodes were harvested. After single-cell suspension, the collected cells from draining lymph nodes were blocked with anti-mouse CD16/32 and stained with Alexa Fluor® 700 anti-mouse CD45, Live/Dead^TM^ Fixable Blue Dead Cell Stain, APC anti-mouse CD11c, FITC anti-mouse CD80 and PE anti-mouse CD86 according to vendor’s protocols. Then cells were analyzed by flow cytometry. Mature DCs were gated as CD45^+^CD11c^+^CD80^+^CD86^+^ cells.

To systemically study the immune response, mice after various therapies were killed, and primary tumors, distant tumors and spleens were harvested and prepared to single cell suspension according to standard protocols. In brief, tumors were minced into tiny pieces and digested with enzyme cocktail (collagenase type I and IV, 1 mg mL^−1^) at 37 °C for 1 h. Thereafter, the mixture was gently grinded and filtered through 70 µm cell strainer to obtain single cell suspension. Spleen was placed in 1× PBS and gently minced with plunger of the syringe. Thereafter, the mixture was filtered through 70 µm cell strainer. Red cells in single-cell suspension were removed by eBioscience^TM^ 1× RBC Lysis Buffer (Invitrogen). In addition, peripheral blood mononuclear cells were isolated by density gradient centrifugation of blood with Histopaque® 1077 (400 × *g*, 30 min). For the analysis of CTL and helper T cell, the collected cells were blocked with anti-mouse CD16/32 and stained with Alexa Fluor® 700 anti-mouse CD45, Live/Dead^TM^ Fixable Blue Dead Cell Stain, FITC anti-mouse CD3, APC anti-mouse CD8 and PE anti-mouse CD4 following the vendor’s protocols. Thereafter, the stained cells were analyzed by flow cytometry.

### Tumor-specific cytotoxicity of CD8^+^ T cells

4T1 or AML-12 (mouse hepatocyte, purchased from ATCC) target cells were seeded in 24-well plate (10^5^ cells per well). Effector cells, which were CD8^+^ T cells were isolated by Dynabeads® Untouched™ Mouse CD8 Cells kit from Balb/c mice after different treatments as described above, were added to target cells. Mixed cells were cultured overnight. Thereafter, viability of target cells was examined by flow cytometry via Live/Dead^TM^ Fixable Blue Dead Cell Stain.

### In vivo photothermal immunotherapy in T-cell-deficient NCr nude mice

To inoculate the primary tumor, 4T1 cells (two million suspended in 100 µL DMEM) was subcutaneously injected into the right flank of female NCr nude mice (Tac:Cr:(Ncr)-Fox1nu, 5 weeks old). Five days later, 4T1 cells (0.5 million cells suspended in 50 µL DMEM) was subcutaneously injected into the left flank of NCr nude mice for secondary tumor inoculation. All other steps were performed in accordance with in vivo photothermal immunotherapy as described above.

### Mechanistic study of photothermal activation in deep tumor

To study the intratumoral temperature, IR thermal camera was placed perpendicularly to the direction of tumor irradiation during in vivo PTT and photothermal immunotherapy. IR thermal images were analyzed by FLIR tools. At day 2 after laser irradiation, mice were killed and tumors were collected and fixed in 4% PFA. After fixation, tumors were dehydrated with 30% sucrose, embedded in optical cutting temperature medium, and sectioned to 10-µm slices on Leica cryostat at different photothermal depths (2, 4, 6, 8 mm). Afterwards, tumor sections were blocked with 3% bovine serum albumin in PBS containing 0.1% Triton X-100 (PBST) for 30 min. Then sections were washed with PBST and respectively incubated with cleaved caspase-3 antibody, HMGB1 antibody, FITC anti-mouse CD80 and PE anti-mouse CD86 in a humidified chamber at 4 °C overnight. After incubation, tumor sections were washed with PBS. For caspase-3 and HMGB1 staining, tumor sections were incubated with secondary antibody Alexa Fluor 488 conjugated donkey anti-rabbit IgG at room temperature for 1 h. After incubation with secondary antibody, these tumor sections were washed with PBS, stained with DAPI and mounted with Fluoromount aquaous mounting medium. After CD80 and CD86 staining, tumor sections were directly washed with PBS, stained with DAPI and mounted with Fluoromount aquaous mounting medium. At last, tumor sections were imaged under confocal microscope. And the quantification of fluorescence intensity was performed by ImageJ. In particular, the expression level of HMGB1 in cytoplasm was calculated as the mean fluorescence intensity of HMGB1 staining in the area without DAPI signal.

### Statistical analysis

Data were presented as mean ± SD unless otherwise stated. Flow cytometry results were analyzed by FlowJo v10. NIR fluorescence images were analyzed by Living Image 4.3 software. Statistical differences were calculated by two-tailed Student’s *t* test using GraphPad Prism 7 or one-way analysis of variance using IBM SPSS Statistics 24. For statistical analysis, *P < 0.05, **P < 0.01, and ***P < 0.001 ****P < 0.0001 were regarded as statistically significant.

### Reporting summary

Further information on research design is available in the Nature Research Reporting Summary linked to this article.

## Supplementary information

Supplementary Information

Reporting Summary

## Data Availability

All the data supporting the findings of this study are available within the article and its supplementary information files and from the corresponding author upon reasonable request. A reporting summary for this article is available as a Supplementary Information file.
