# Supplementary material for: Activatable polymer nanoagonist for second near-infrared photothermal immunotherapy of cancer
Source: Nat Commun. 2021 Feb 2;12:742. doi: 10.1038/s41467-021-21047-0 (PMC7854754; doi:10.1038/s41467-021-21047-0)
Supplement: Supplementary file 1 — Supplementary Information [file 41467_2021_21047_MOESM1_ESM.pdf]

Supplementary Information for

Activatable polymer nano-agonist for second near-infrared photothermal immunotherapy of cancer

*Yuyan Jiang[1], Jiaguo Huang[1], Cheng Xu[1], and Kanyi Pu[1, 2]**

[1]School of Chemical and Biomedical Engineering, Nanyang Technological University, 70 Nanyang Drive, Singapore 637457, Singapore.

[2]Division of Chemistry and Biological Chemistry, School of Physical and Mathematical Sciences, Nanyang Technological University, 21 Nanyang Link, 637371, Singapore.

*Corresponding author. Email: kypu@ntu.edu.sg

This PDF file includes:

Supplementary Figure 1. Absorption spectrum of pBODO-Br in THF.

Supplementary Figure 2. GPC result of pBODO-Br in THF.

Supplementary Figure 3. ^{1}H NMR spectrum of pBODO-N_3 in $CDCl_3$.

Supplementary Figure 4. ^{1}H NMR spectrum of VCOOH in CD_3OD.

Supplementary Figure 5. ^{1}H NMR spectrum of VR in CD_3OD.

Supplementary Figure 6. ^{1}H NMR spectrum of alkyne-PEG-NH_2 in $CDCl_3$.

Supplementary Figure 7. ^{1}H NMR spectrum of alkyne-PEG-VR in $CDCl_3$.

Supplementary Figure 8. ^{1}H NMR spectrum of pBODO-PEG-VR in $CDCl_3$.

Supplementary Figure 9. (a) Zeta potential files of APNA and APNC. (b) DLS profiles of APNA and APNC in 1 × PBS for 2 months.

Supplementary Figure 10. Measurement of photothermal conversion efficiency of APNC.

Supplementary Figure 11. ^{1}H NMR spectrum of activated agonist (Fig. 2e) after photothermal activation of APNA in CD_3OD.

Supplementary Figure 12. LCMS spectrum of photothermally activated agonist.

Supplementary Figure 13. LCMS spectrum of final product (R848) after hydrolysis of activated agonist by esterase.

Supplementary Figure 14. Fluorescence spectra of APNA or APNC ([pBODO] = 2 µg mL^{-1}) in 1 × PBS. Excitation: 450 nm.

Supplementary Figure 15. (a) Absorption and (b) fluorescence spectra of $APNA_F$ and $APNC_F$.

Supplementary Figure 16. Quantification of NIR fluorescence intensity (assigned to SPN) of 4T1 cells or BMDCs in Fig. 3a and Fig.3b.

Supplementary Figure 17. Gating strategies for flow cytometry analysis of immune cells in (a) lymph node, (b) spleen, (c) tumor, (d) blood and (e) in vitro tumor-specific cytotoxicity assay.

Supplementary Figure 18. In vitro T cell stimulation capacity of DCs after various treatments.

Supplementary Figure 19. In vivo fluorescence imaging.

Supplementary Figure 20. Body weights of living mice during various treatments in Fig. 4 and 5.

Supplementary Figure 21. H&E images of major organs from mice after various therapies.

Supplementary Figure 22. Flow cytometry analysis of immune cells in blood.

Supplementary Figure 23. In vivo immune responses in spleen after NIR-II photothermal immunotherapy.

Supplementary Figure 24. Detection of tumor-specific cytotoxicity of $CD8^+$ T cells from living mice after various treatments.

Supplementary Figure 25. In vivo NIR-II photothermal immunotherapy in T cell-deficient NCr nude mice.

Supplementary Figure 26. Immunofluorescent images of Cas-3 (green fluorescence) in tumor sections at different photothermal depths at day 2 after various treatments.

Supplementary Figure 27. Immunofluorescent images of HMGB1 (green fluorescence) in tumor sections at different photothermal depths at day 2 after various treatments.

Supplementary Figure 28. Immunofluorescent images of CD80 (green fluorescence) and CD86 (orange fluorescence) in tumor sections at different photothermal depths at day 2 after various treatments.

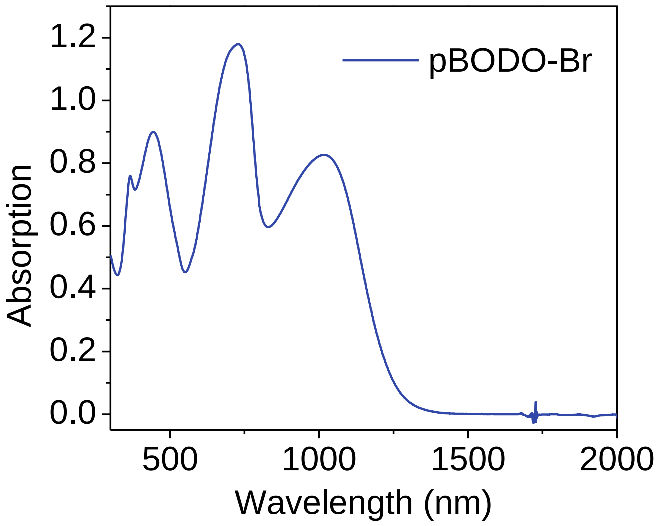

Supplementary Figure 1. Absorption spectrum of pBODO-Br in THF.

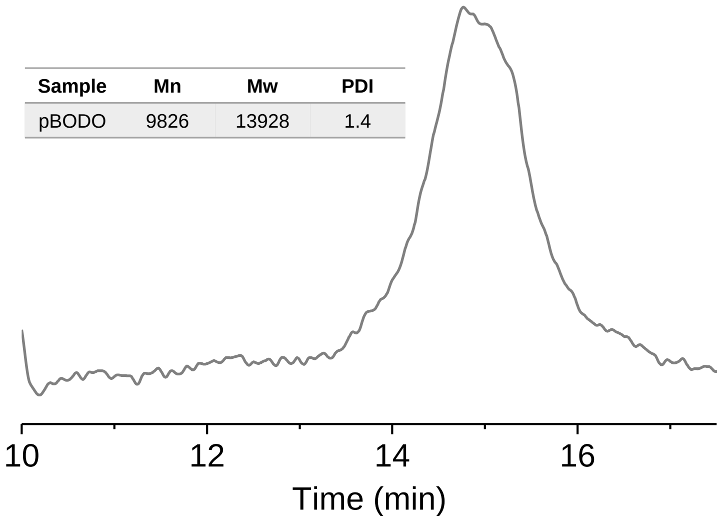

Sample	Mn	Mw	PDI
pBODO	9826	13928	1.4

Supplementary Figure 2. GPC result of pBODO-Br in THF. PDI, polydispersity index.

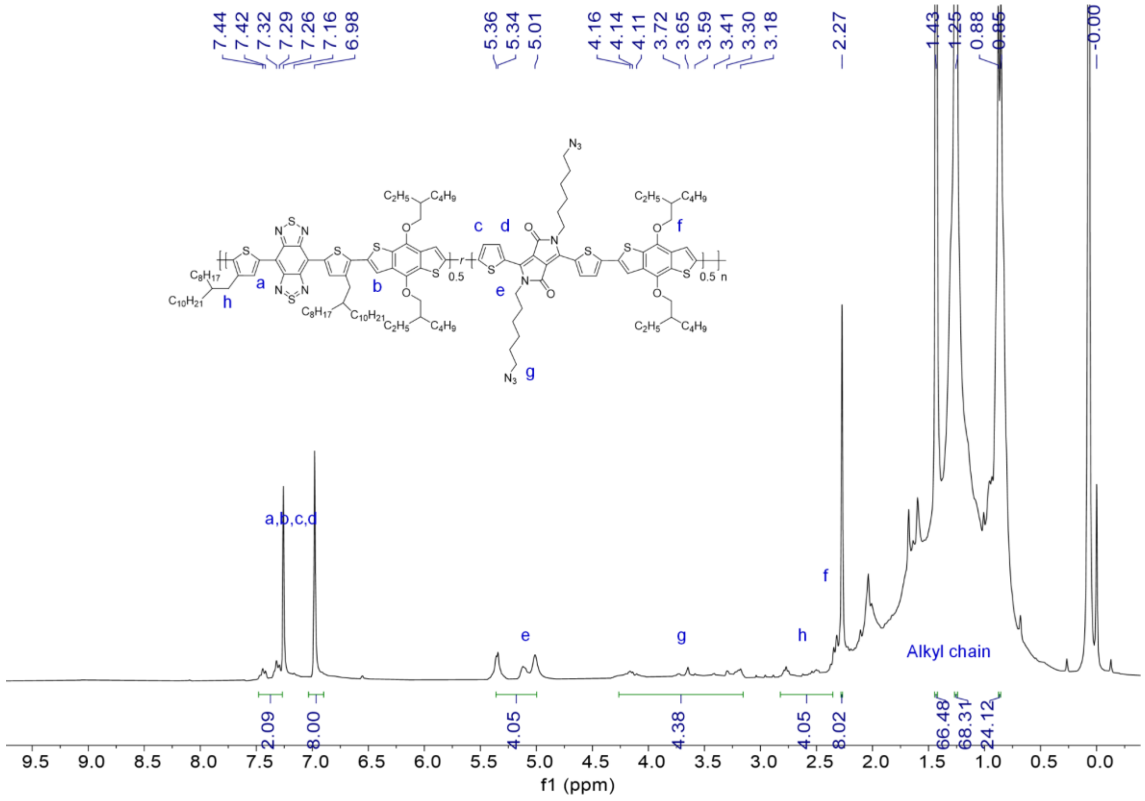

Supplementary Figure 3. **^{1}H NMR spectrum of pBODO-N_3 in $CDCl_3$.**

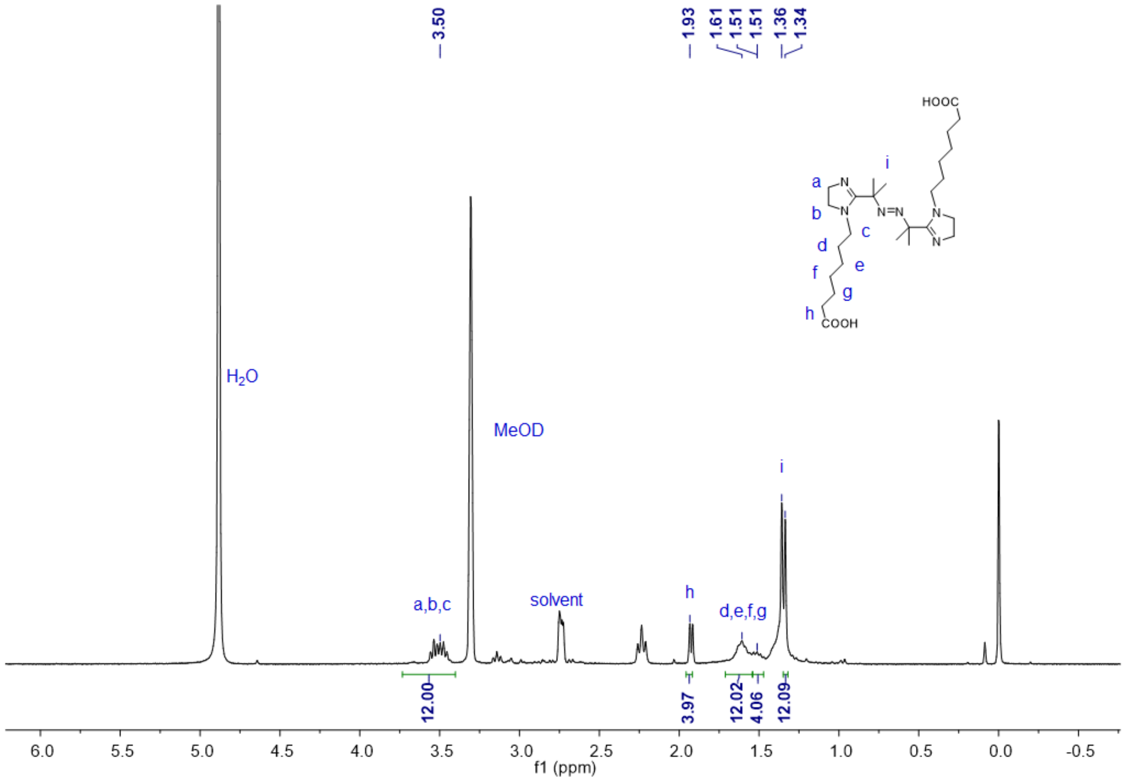

Supplementary Figure 4. **^{1}H NMR spectrum of VCOOH in CD_3OD.**

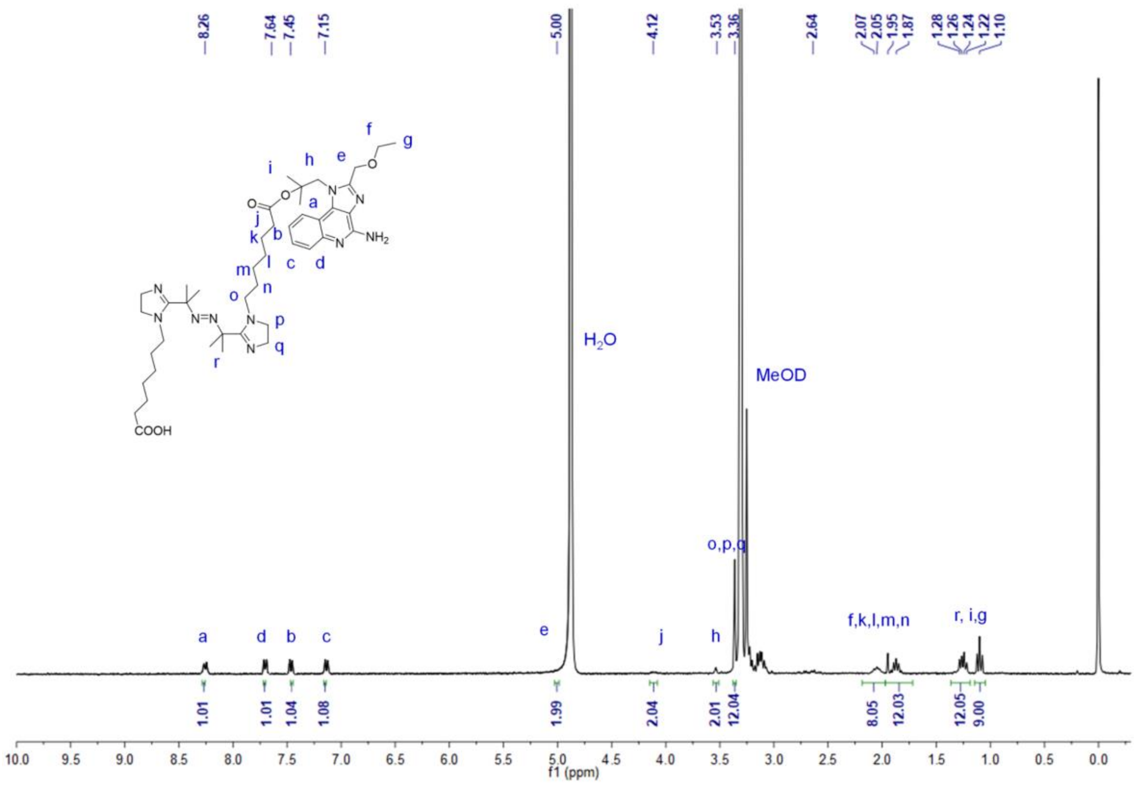

Supplementary Figure 5. **^{1}H NMR spectrum of VR in CD_3OD.**

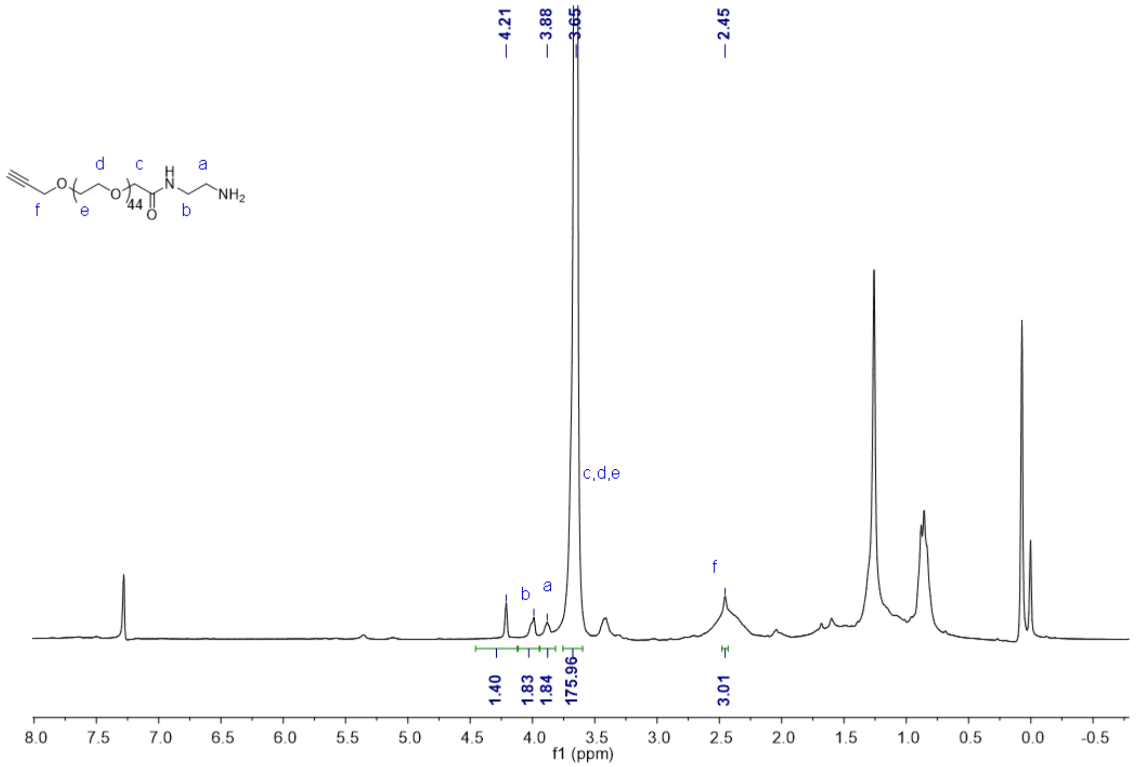

Supplementary Figure 6. **^{1}H NMR spectrum of alkyne-PEG-NH_2 in $CDCl_3$.**

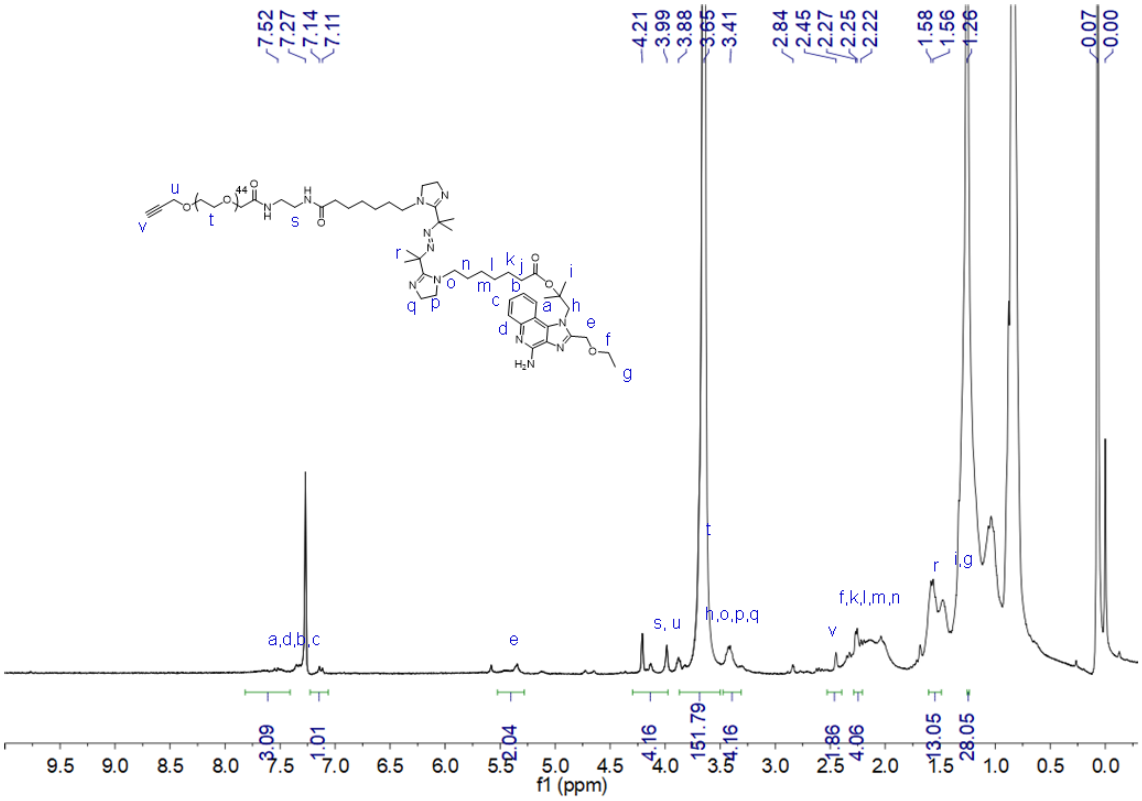

Supplementary Figure 7. ^{1}H NMR spectrum of alkyne-PEG-VR in $CDCl_3$.

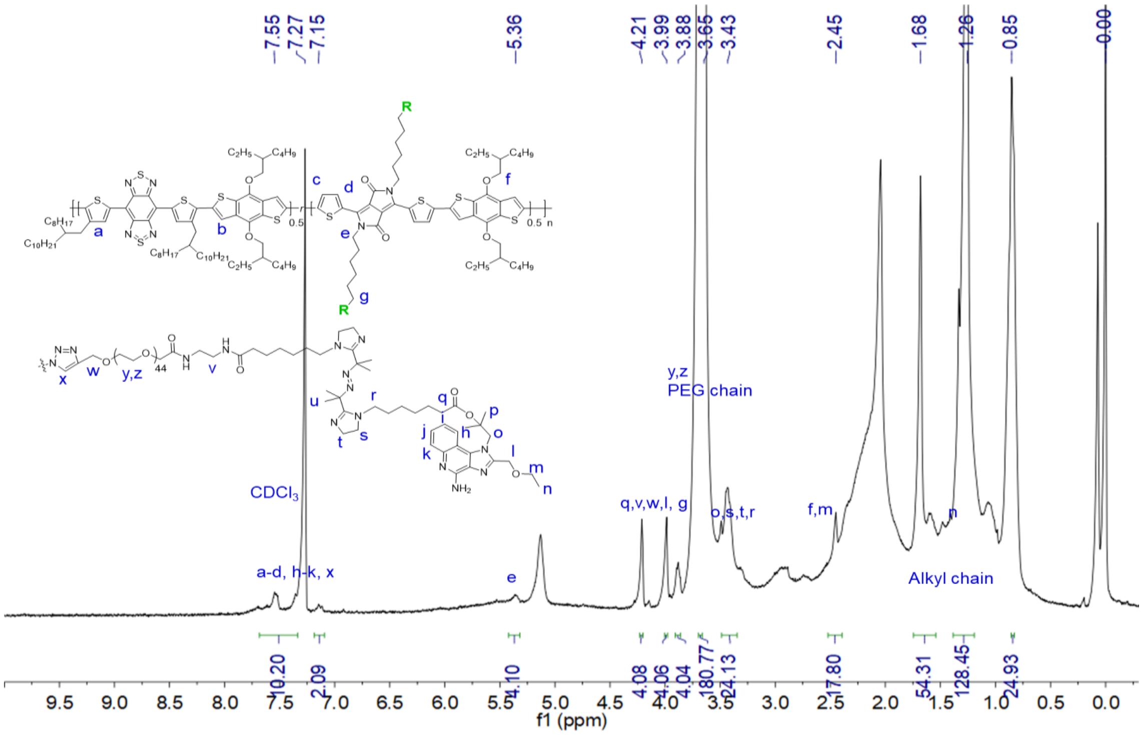

Supplementary Figure 8. ^{1}H NMR spectrum of pBODO-PEG-VR in $CDCl_3$.

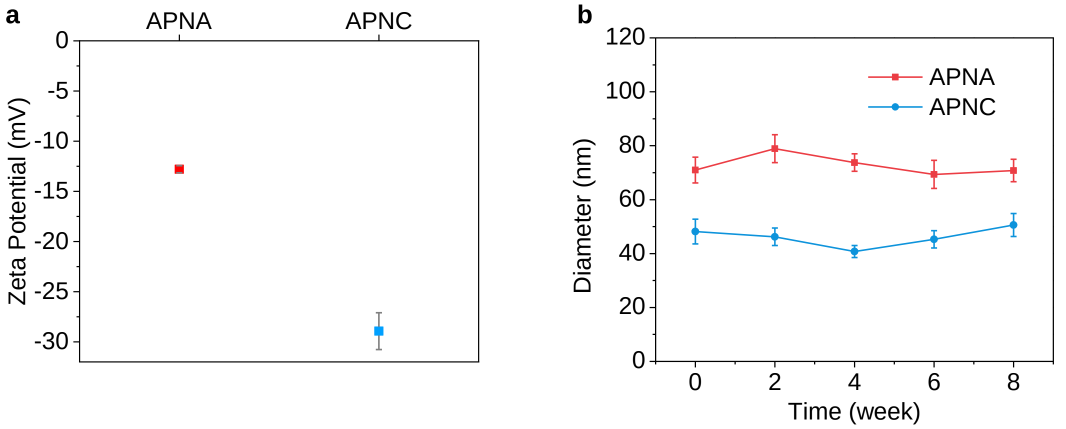

Supplementary Figure 9. (a) Zeta potential files of APNA and APNC. (b) DLS profiles of APNA and APNC in 1 × PBS for 2 months. Data were expressed as mean ± SD. Error bars indicated standard deviations of three independent measurements.

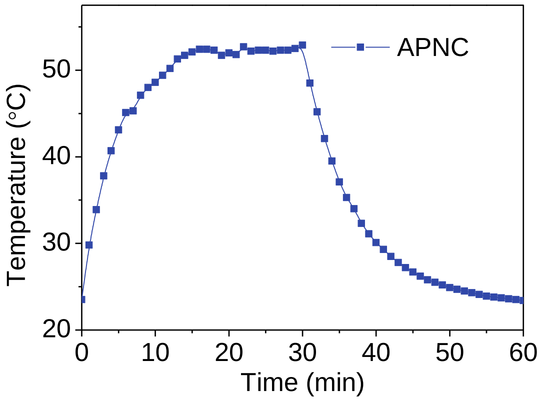

Supplementary Figure 10. Measurement of photothermal conversion efficiency of APNC. APNC solution (optical density at 1064 nm = 1, 2 mL) was irradiated with 1064 nm laser (1 W cm^{-2}) for 30 min followed by natural cooling for another 30 min.

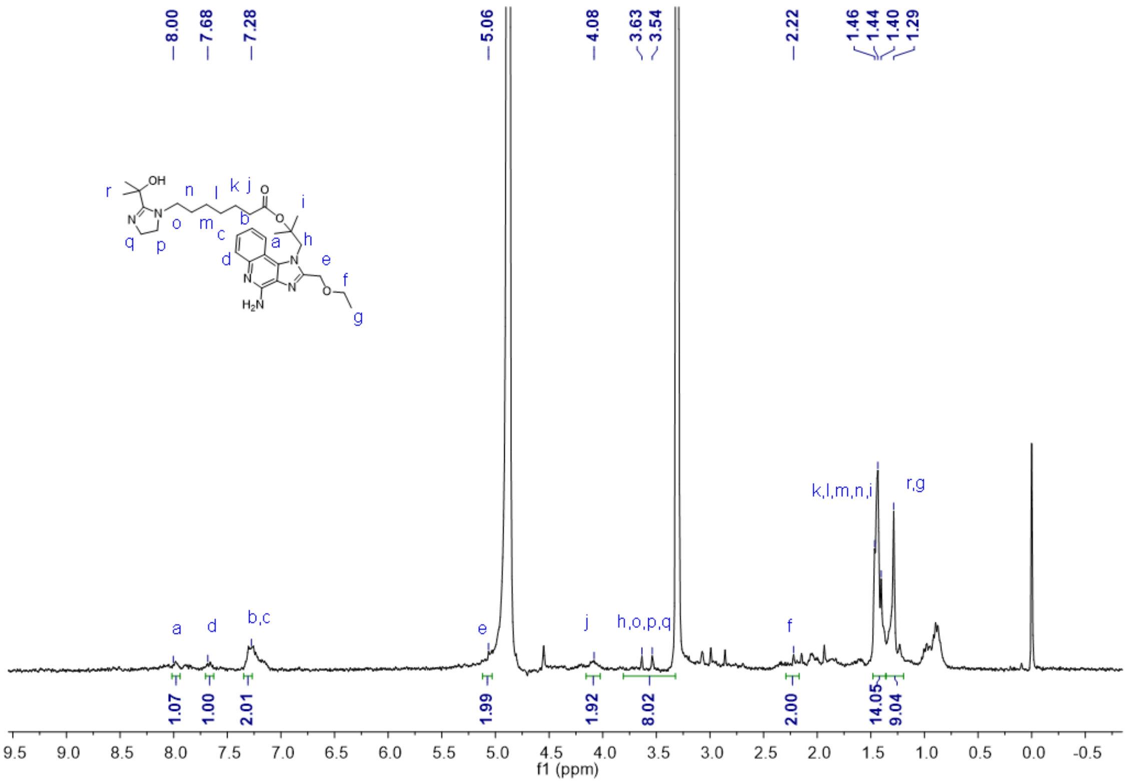

Supplementary Figure 11. ^{1}H NMR spectrum of activated agonist (Fig. 2E) after photothermal activation of APNA in CD_3OD. The activated agonist was obtained by photoirradiation (1 W cm^{-2}, 10 min) of APNA solution (20 μg mL^{-1}, 200 μL), followed by filtration through 220 nm filter and purification by HPLC (T_R = 24.7 min).

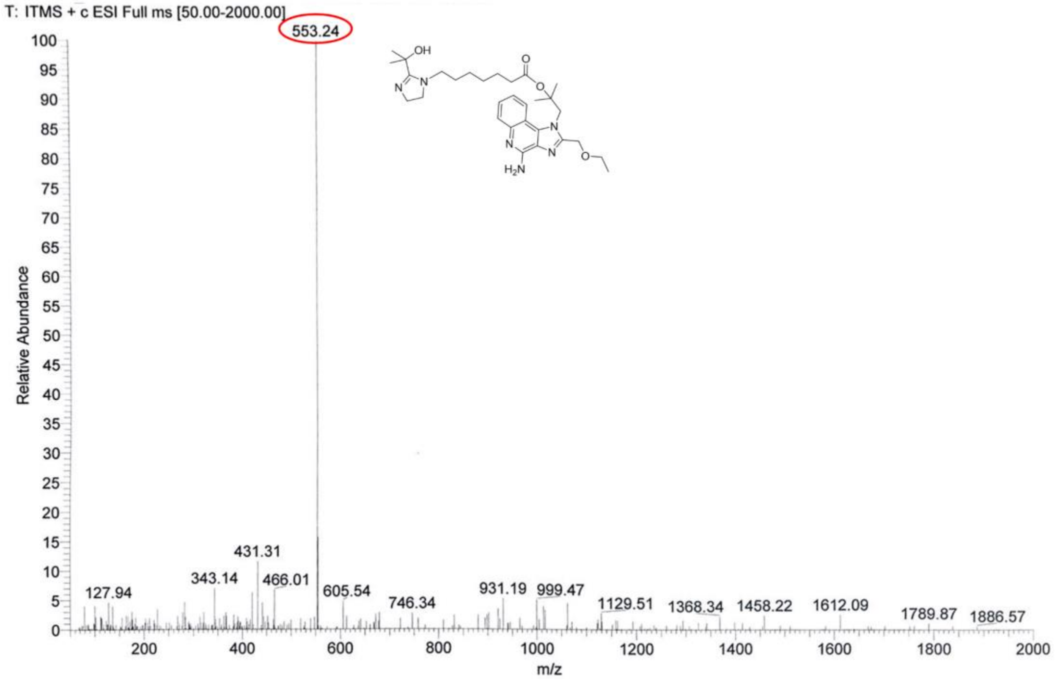

Supplementary Figure 12. LCMS spectrum of photothermally activated agonist.

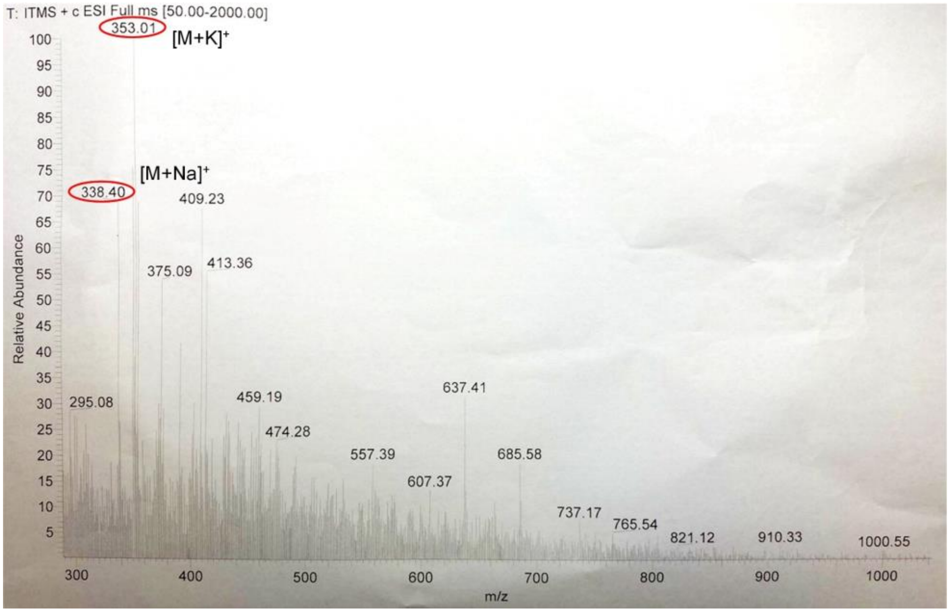

Supplementary Figure 13. LCMS spectrum of final product (R848) after hydrolysis of activated agonist by esterase.

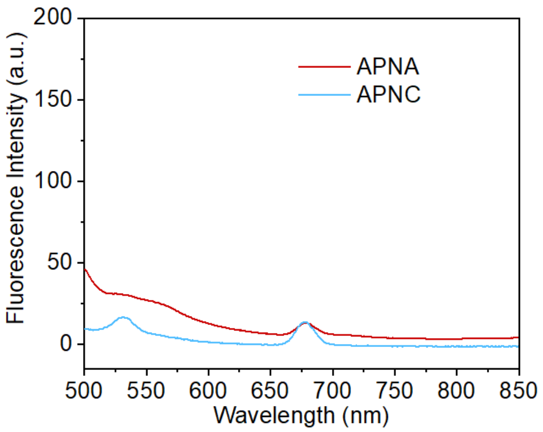

Supplementary Figure 14. Fluorescence spectra of APNA or APNC ([pBODO] = 2 μg mL^{-1}) in 1 × PBS. Excitation: 450 nm.

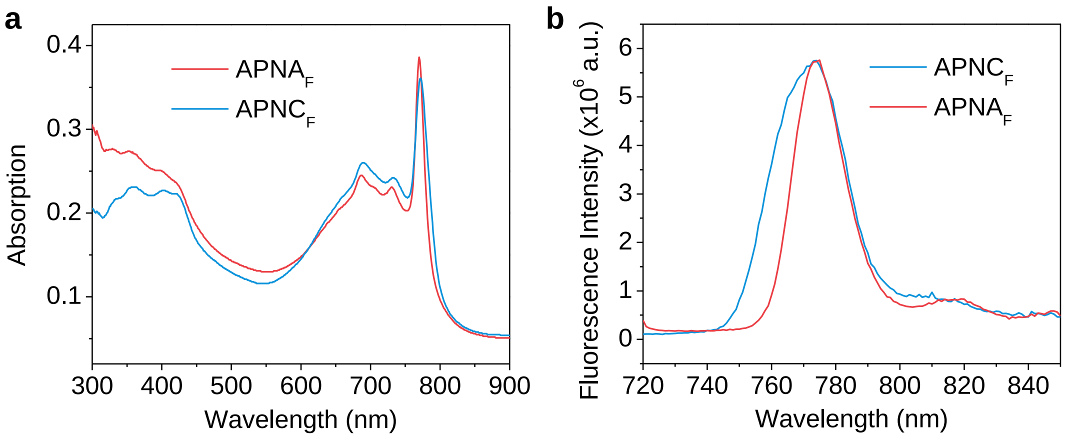

Supplementary Figure 15. Absorption (a) and fluorescence (b) spectra of APNA$_F$ and APNC$_F$ in 1 × PBS. [pBODO] = 10 μg mL^{-1}.

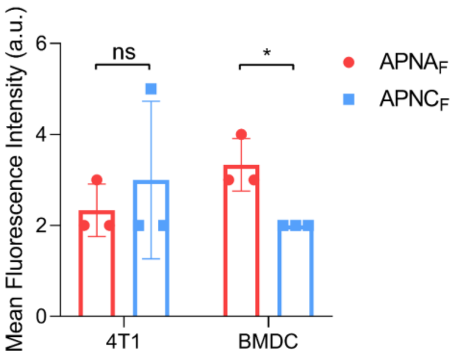

Supplementary Figure 16. Quantification of NIR fluorescence intensity (assigned to SPN) of 4T1 cells or BMDCs in Fig. 3a and Fig.3b. Cells were cultured with APNA$_F$ or APNC$_F$

([pBODO] = 10 µg Ml^{-1}, 2.5 w/w% NCBS) for 24 before measurement (n = 3). Data were expressed as mean ± SD. Error bars indicated standard deviations of three independent measurements. 4T1: P = 0.5614; BMDC: P = 0.0161. Statistical analysis was performed by two-tailed Student's t-test. ns: not significant; *P < 0.05, **P < 0.01; ***P < 0.001; ****P < 0.0001.

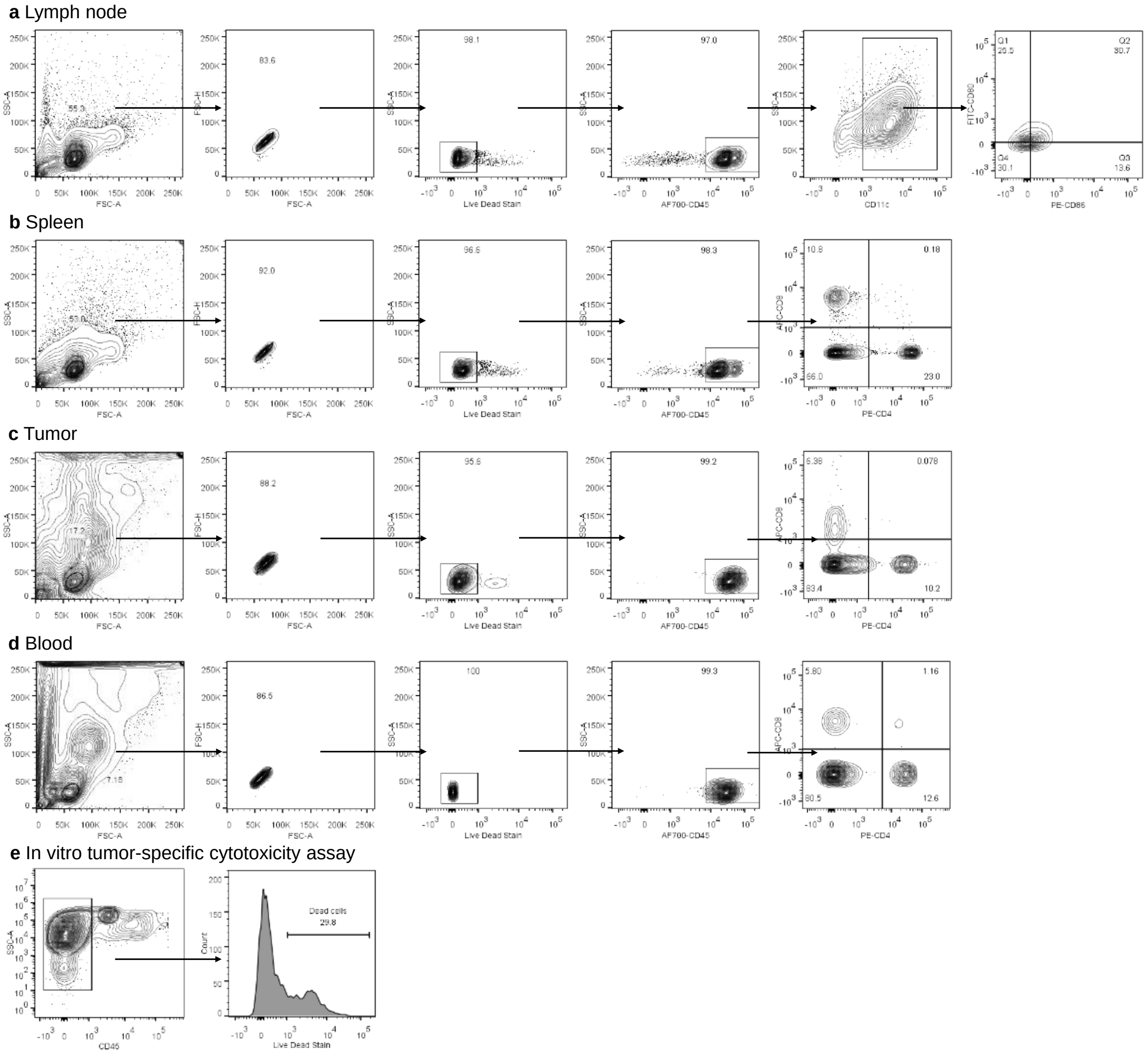

Supplementary Figure 17. Gating strategies for flow cytometry analysis of immune cells in (a) lymph node, (b) spleen, (c) tumor, (d) blood and (e) in vitro tumor-specific cytotoxicity assay.

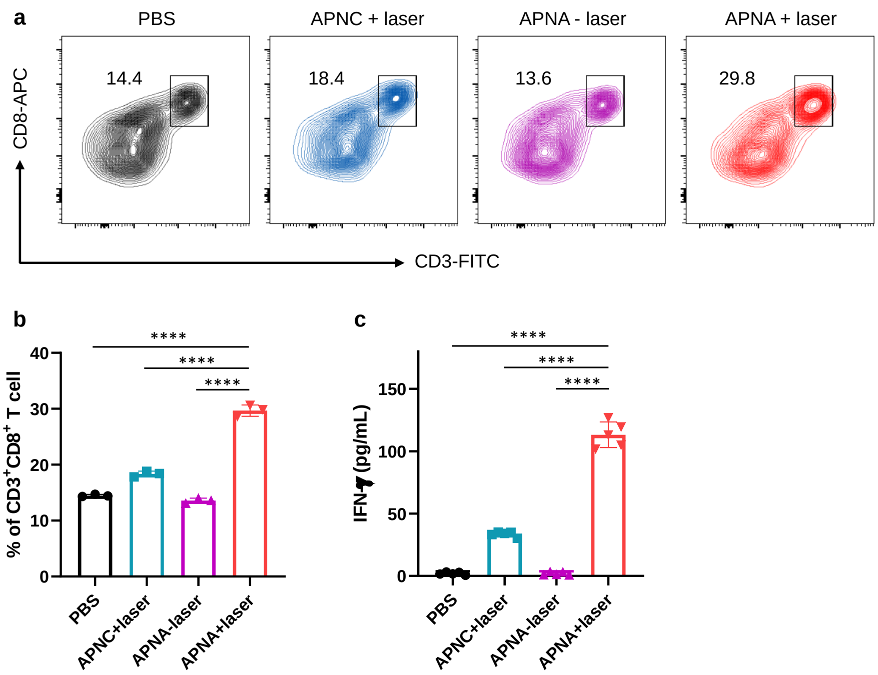

Supplementary Figure 18. In vitro T cell stimulation capacity of DCs after various treatments. (a) Flow cytometry plots of the percentage of $CD3^{+}CD8^{+}$ T cells after incubation of naïve T cells with stimulated DCs. DCs were pre-treated for 48 h with PBS, photo-irradiated APNC, APNA, photo-irradiated APNA, respectively. [Pbodo] = 10 μg Ml^{-1}; 1064 nm photoirradiation: 1 W cm^{-2}, 6 min. (b) Quantification of percentage of $CD3^{+}CD8^{+}$ T cells in mixed lymphocytes in (a) (n = 3). $P < 0.0001$ (c) IFN-ɣ levels in the supernatants of various mixed lymphocytes in (a) (n = 5). $P < 0.0001$. Data were expressed as mean ± SD. Statistical analysis was performed by two-tailed Student's *t*-test. Ns: not significant; $^{*}P < 0.05$, $^{**}P < 0.01$ and $^{***}P < 0.001$.

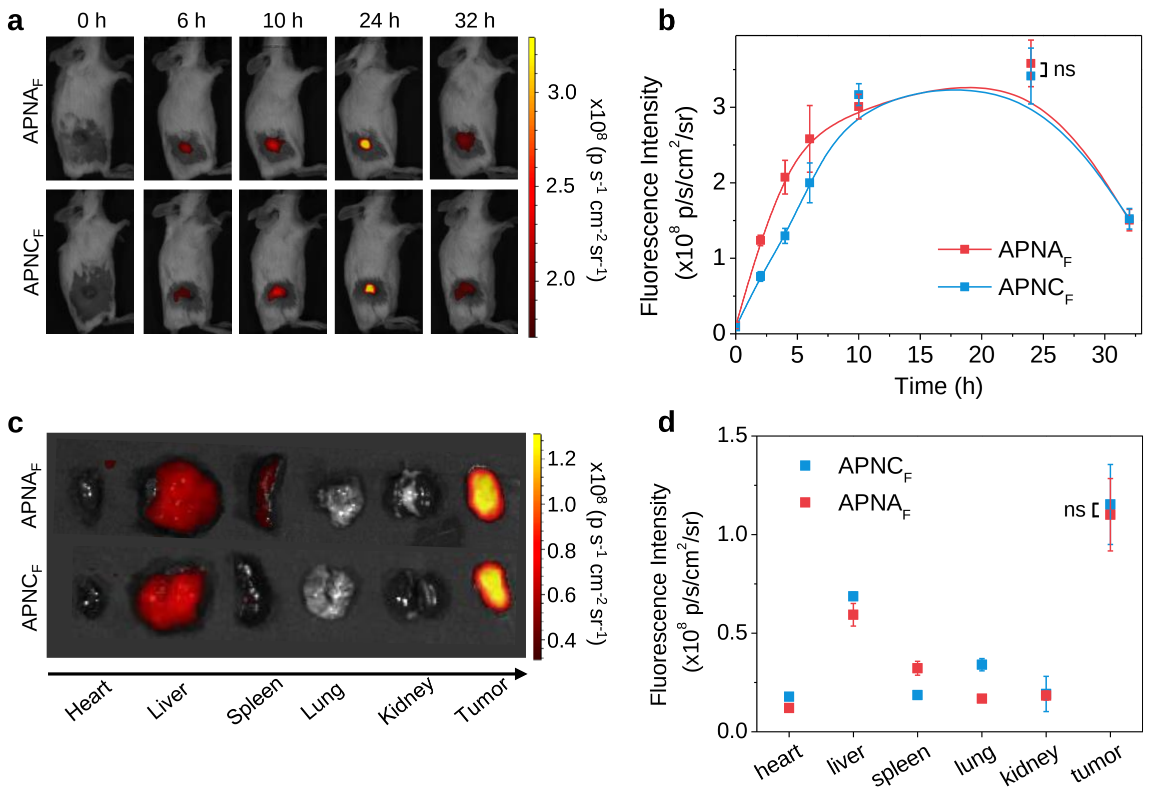

Supplementary Figure 19. In vivo NIR fluorescence imaging. (a) NIR fluorescence images of 4T1-tumor bearing Balb/c mice at different time points after intravenous injection of $APNA_F$ or $APNC_F$ (200 μL per mouse, [Pbodo] = 250 μg Ml^{-1}). NIR fluorescence was acquired with excitation at 710 nm and emission at 780 nm. (b) Quantification of fluorescence signals in (a) (n=3). (c) NIR fluorescence images of major organs and tumors from mice at 32 h post-injection of nanoparticles. (d) Quantification of NIR fluorescence signals in (c) (n=3). Statistical analysis was performed by one-way ANOVA analysis. *$P < 0.05$, **$P < 0.01$ and ***$P < 0.001$.

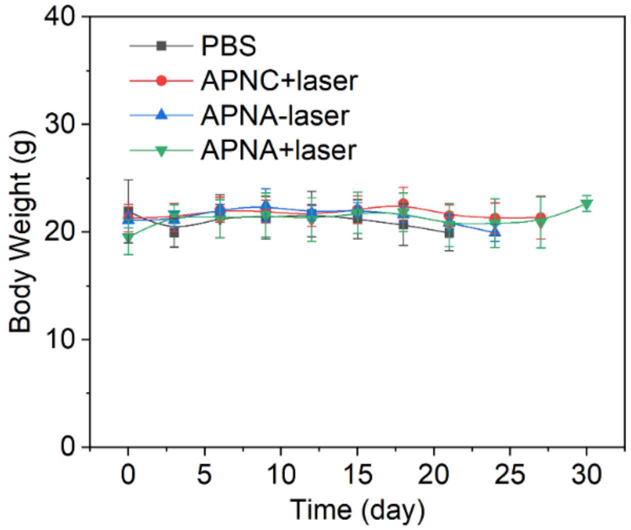

Supplementary Figure 20. Body weights of living mice during various treatments in Fig. 4 and 5 (n = 6). Data were expressed as mean ± SD.

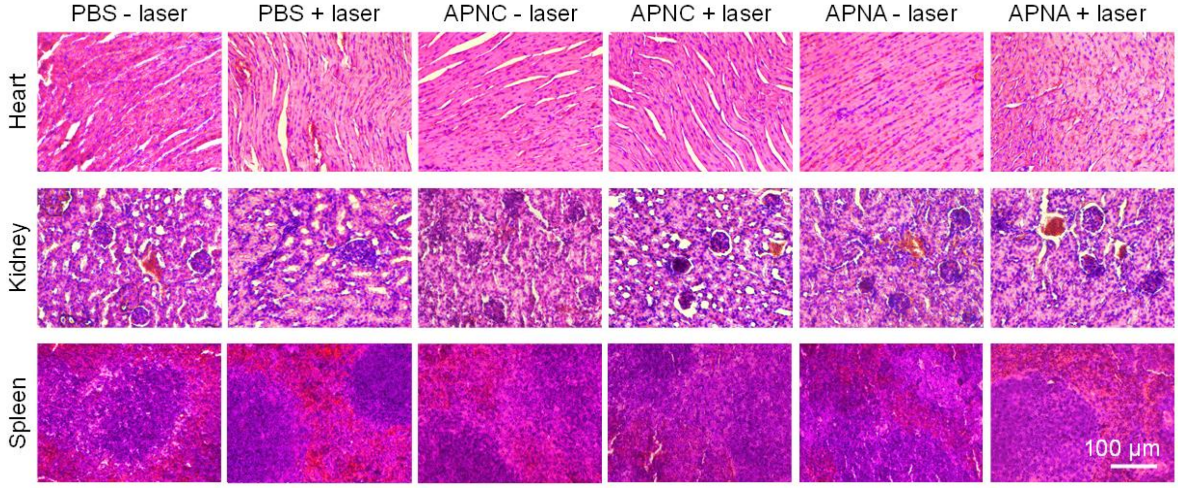

Supplementary Figure 21. H&E images of major organs from mice after various therapies. Hearts, kidneys, and spleens were harvested from mice at day 14 after intravenous administration of saline (200 μL per mouse), APNA or APNC (200 μL per mouse, [pBODO] = 250 μg mL^{-1}) with or without 1064 nm photoirradiation (1 W cm^{-2}, 10 min). Experiments were performed in triplicate with similar results.

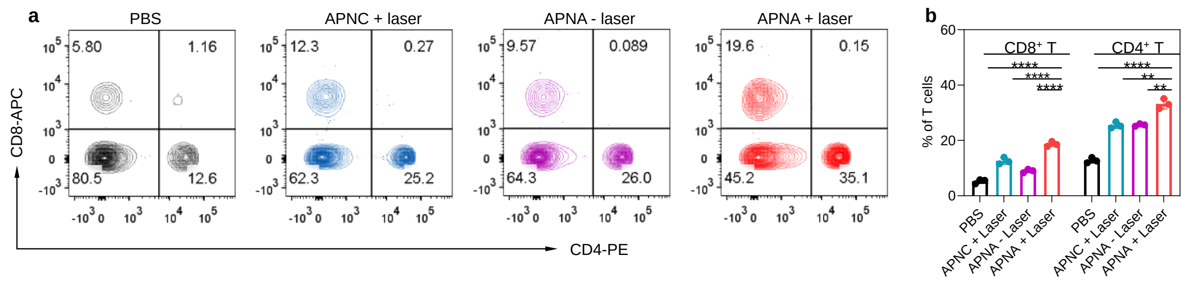

Supplementary Figure 22. Flow cytometry analysis of immune cells in blood. (a) Representative flow cytometry plots showing $CD8^+$ T cells and $CD4^+$ T cells in the blood from mice (gated on $CD45^+$ lymphocytes) after various treatments. (b) Quantification of $CD8^+$ T cells and $CD4^+$ T cells as a percentage of $CD45^+$ lymphocytes in the blood (n = 3). For $CD8^+$ T cells, $P < 0.0001$. For $CD4^+$ T cells: PBS + Laser and APNA + Laser: $P < 0.0001$; APNC + Laser and APNA + Laser: $P = 0.0033$; APNA - Laser and APNA + Laser: $P = 0.0021$. Data were expressed as mean ± SD. Statistical analysis was performed by two-tailed Student's *t*-test. ns: not significant; $^*P < 0.05$; $^{**}P < 0.01$; $^{***}P < 0.001$; $^{****}P < 0.0001$.

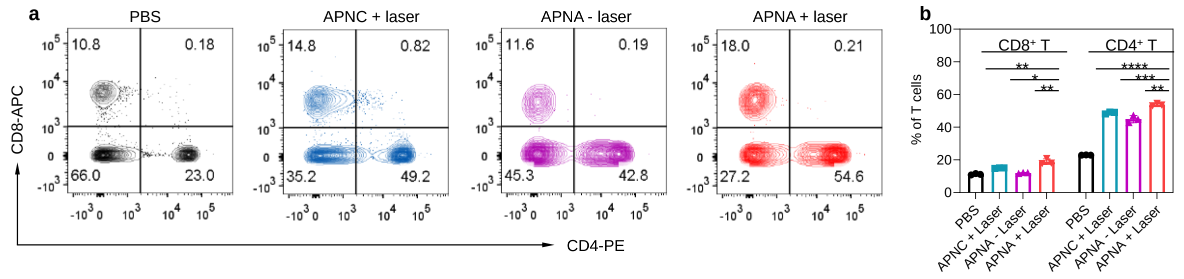

Supplementary Figure 23. In vivo immune responses in spleen after NIR-II photothermal immunotherapy. (a) Representative flow cytometry plots showing $CD8^+$ T cells and $CD4^+$ T cells in spleens from mice (gated on $CD45^+$ lymphocytes) after various treatments. (b) Quantification of $CD8^+$ T cells and $CD4^+$ T cells as a percentage of $CD45^+$ lymphocytes in spleens (n = 3). For $CD8^+$ T cells, PBS + Laser and APNA + Laser: $P = 0.0017$; APNC + Laser and APNA + Laser: $P = 0.0179$; APNA - Laser and APNA + Laser: $P = 0.0024$. For $CD4^+$ T cells: PBS + Laser and APNA + Laser: $P < 0.0001$; APNC + Laser and APNA + Laser: $P = 0.0009$; APNA - Laser and APNA + Laser: $P = 0.0028$. Data were expressed as mean ± SD.

Statistical analysis was performed by two-tailed Student's *t*-test. ns: not significant; **P* < 0.05; ***P* < 0.01; ****P* < 0.001; *****P* < 0.0001.

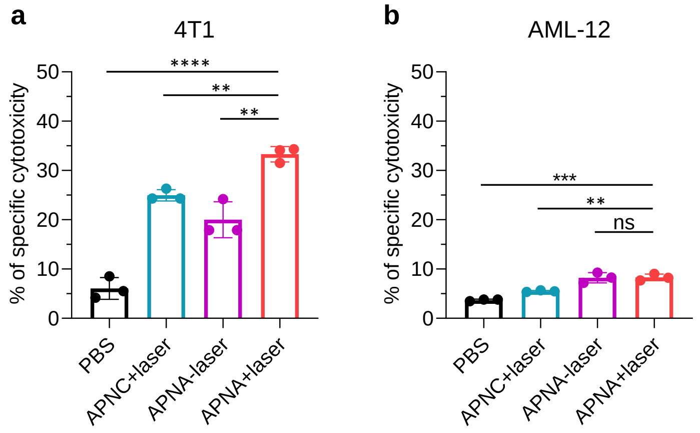

Supplementary Figure 24. Detection of tumor-specific cytotoxicity of CD8⁺ T cells from living mice after various treatments. After various anti-cancer treatments, $CD8^+$ T cells were isolated from mice by Dynabeads® Untouched™ Mouse CD8 Cells kit and then co-cultured with various cell lines. (a) Percentage of specific cytotoxicity to 4T1 cells. PBS + Laser and APNA + Laser: *P* <0.0001; APNC + Laser and APNA + Laser: *P* = 0.0018; APNA - Laser and APNA + Laser: *P* = 0.0043. (b) Percentage of specific cytotoxicity to AML-12 cells (murine hepatocyte cell line). PBS + Laser and APNA + Laser: *P* = 0.0003; APNC + Laser and APNA + Laser: *P* = 0.002; APNA - Laser and APNA + Laser: *P* = 0.9086. Experiments were conducted in triplicate. Data were expressed as mean ± SD. Statistical analysis was performed by two-tailed Student's *t*-test. ns: not significant; **P* < 0.05, ***P* < 0.01, ****P* < 0.001, *****P* < 0.001.

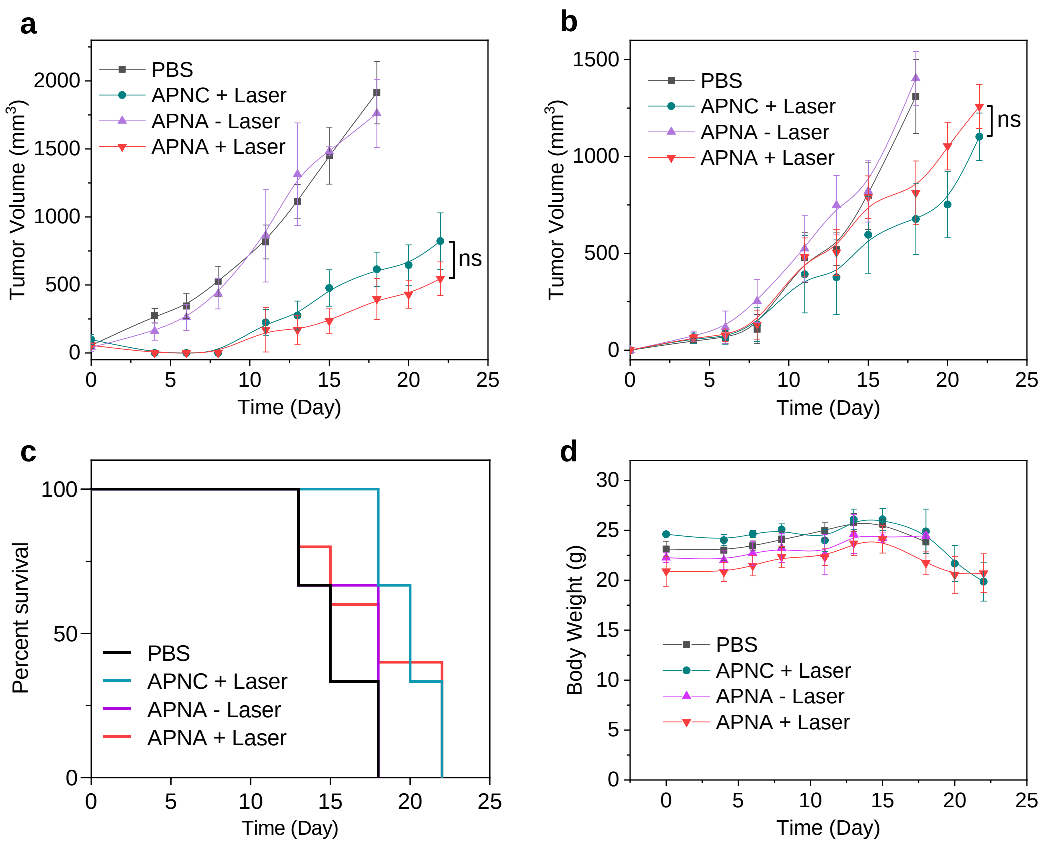

Supplementary Figure 25. In vivo NIR-II photothermal immunotherapy in T cell-deficient NCr nude mice. In accordance with in vivo immunotherapy on Balb/c mice (Fig. 4), after inoculation of both primary and distant tumors, NCr nude mice were respectively i.v. injected with PBS (200 μL per mouse), APNC or APNA (pBODO] = 250 μg mL^{-1}, 200 μL per mouse) (n = 5), followed by photoirradiation (1064 nm, 1 W cm^{-2}, 10 min) to several groups at 24 h post-injection. (a-b) Tumor volumes of primary (a) and distant (b) tumors in NCr nude mice after various treatments (n = 5). (c) Survival curves of 4T1-tumor bearing NCr nude mice after various treatments. (d) Body weights of 4T1-tumor bearing NCr nude mice after various treatments (n = 5). Data were expressed as mean ± SD. Statistical significance was determined by two-tailed Student's *t*-test. ns: not significant; **P* < 0.05.

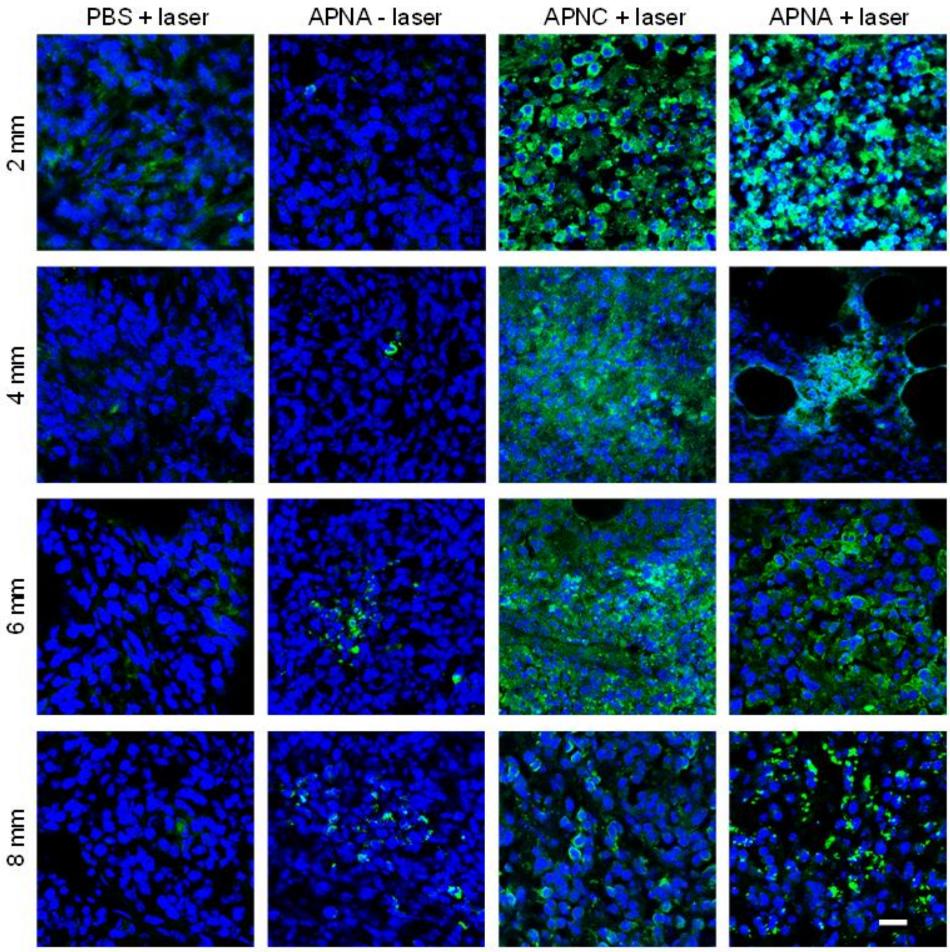

Supplementary Figure 26. Immunofluorescent images of Cas-3 (green fluorescence) in tumor sections at different photothermal depths at day 2 after various treatments. Blue fluorescence indicated nuclei staining by DAPI. Scale bar: 20 μm. Experiments were performed in triplicate with similar results.

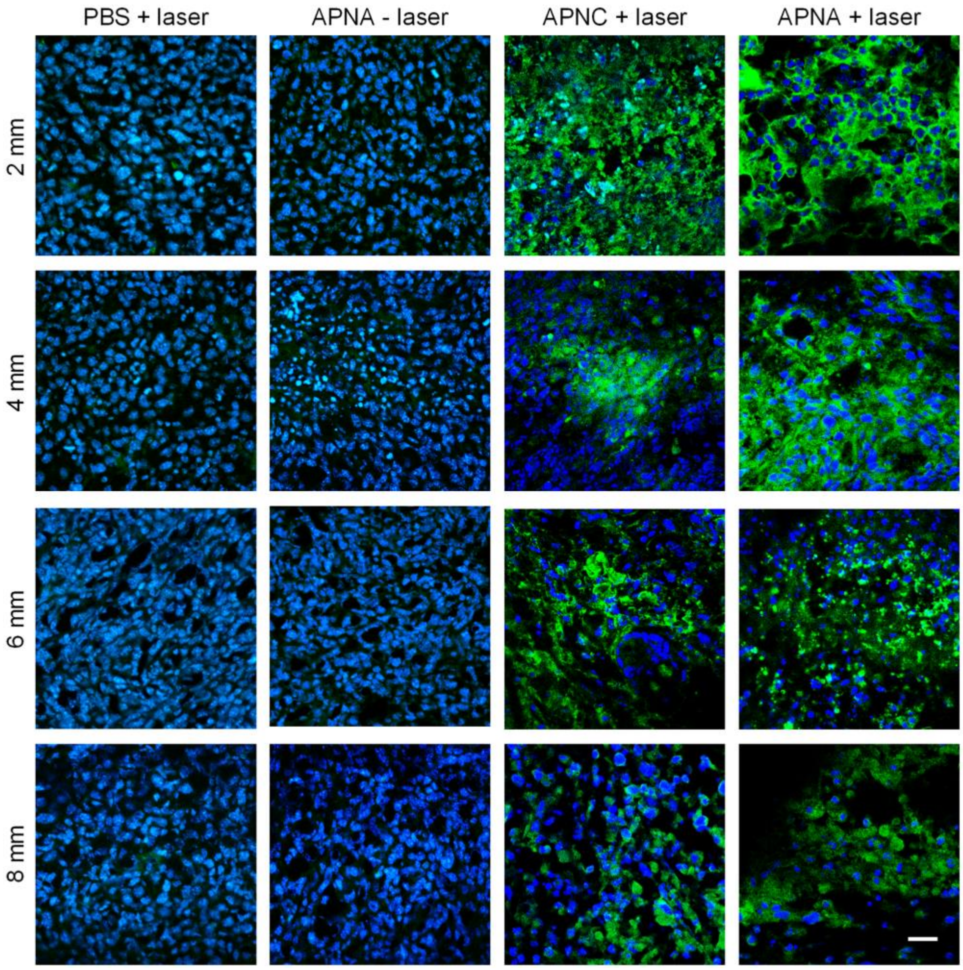

Supplementary Figure 27. Immunofluorescent images of HMGB1 (green fluorescence) in tumor sections at different photothermal depths at day 2 after various treatments. Blue fluorescence indicated nuclei staining by DAPI. Scale bar: 20 μm. Experiments were performed in triplicate with similar results.

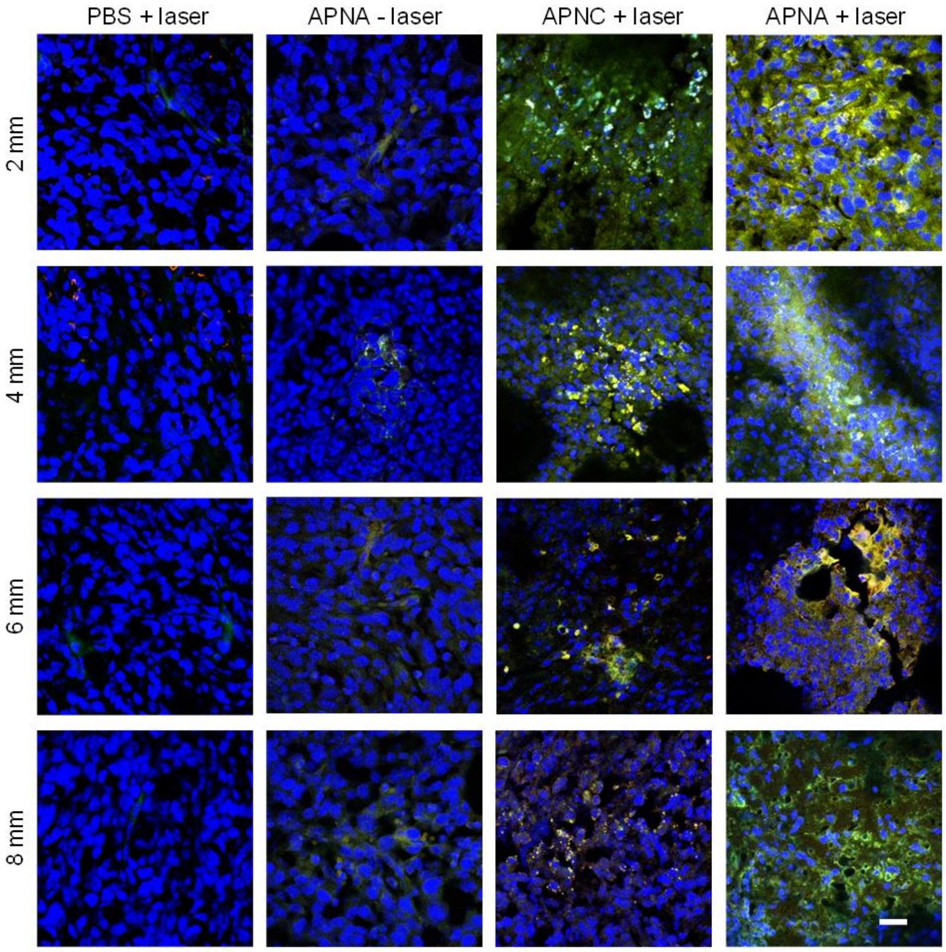

Supplementary Figure 28. Immunofluorescent images of CD80 (green fluorescence) and CD86 (orange fluorescence) in tumor sections at different photothermal depths at day 2 after various treatments. Blue fluorescence indicated nuclei staining by DAPI. Scale bar: 20 μm. Experiments were performed in triplicate with similar results.
