# Supplementary material for: Activatable polymer nanoagonist for second near-infrared photothermal immunotherapy of cancer
Source: Nat Commun. 2021 Feb 2;12:742. doi: 10.1038/s41467-021-21047-0 (PMC7854754; doi:10.1038/s41467-021-21047-0)
Supplement: Supplementary file 2 — Reporting Summary [file 41467_2021_21047_MOESM2_ESM.pdf]

nature research

Corresponding author(s): Kanyi Pu

Last updated by author(s): Oct 22, 2020

Reporting Summary

Nature Research wishes to improve the reproducibility of the work that we publish. This form provides structure for consistency and transparency in reporting. For further information on Nature Research policies, see our Editorial Policies and the Editorial Policy Checklist.

Statistics

For all statistical analyses, confirm that the following items are present in the figure legend, table legend, main text, or Methods section.

n/a	Confirmed	
☐	☒	The exact sample size (n) for each experimental group/condition, given as a discrete number and unit of measurement
☐	☒	A statement on whether measurements were taken from distinct samples or whether the same sample was measured repeatedly
☐	☒	The statistical test(s) used AND whether they are one- or two-sided<br>*Only common tests should be described solely by name; describe more complex techniques in the Methods section.*
☐	☒	A description of all covariates tested
☐	☒	A description of any assumptions or corrections, such as tests of normality and adjustment for multiple comparisons
☐	☒	A full description of the statistical parameters including central tendency (e.g. means) or other basic estimates (e.g. regression coefficient) AND variation (e.g. standard deviation) or associated estimates of uncertainty (e.g. confidence intervals)
☐	☒	For null hypothesis testing, the test statistic (e.g. F, t, r) with confidence intervals, effect sizes, degrees of freedom and P value noted<br>*Give P values as exact values whenever suitable.*
☒	☐	For Bayesian analysis, information on the choice of priors and Markov chain Monte Carlo settings
☒	☐	For hierarchical and complex designs, identification of the appropriate level for tests and full reporting of outcomes
☒	☐	Estimates of effect sizes (e.g. Cohen's d, Pearson's r), indicating how they were calculated

Our web collection on statistics for biologists contains articles on many of the points above.

Software and code

Policy information about availability of computer code

Data collection	Irrelevant to experiments.
Data analysis	Fluorescence and afterglow images were analyzed using the Living Image 4.3 Software (PerkinElmer). NMR spectra were analyzed using Mestre Nova LITE v5.2.5-4119 software (Mestre lab Research S.L.).

For manuscripts utilizing custom algorithms or software that are central to the research but not yet described in published literature, software must be made available to editors and reviewers. We strongly encourage code deposition in a community repository (e.g. GitHub). See the Nature Research guidelines for submitting code & software for further information.

Data

Policy information about availability of data

All manuscripts must include a data availability statement. This statement should provide the following information, where applicable:

- Accession codes, unique identifiers, or web links for publicly available datasets
- A list of figures that have associated raw data
- A description of any restrictions on data availability

The authors declare that all related to this study are available in the article/and or its supplementary information files.

Field-specific reporting

Life sciences study design

All studies must disclose on these points even when the disclosure is negative.

Sample size	We used G*power analysis to calculate and ensure the sample sizes fulfill adequate power (p>0.8). According to the experimental data and sample size (n), P value and effect size were calculated and the power was then calculated. If it is more than 80%, demonstrating the sample size is adequate.
Data exclusions	No data was excluded from this study.
Replication	Experiments were repeated at least three independent experiments with similar results. All experiments were reproduced to reliably support conclusions stated in the manuscript.
andomization	Cages of mice were randomly selected and then divided into experimental groups for further treatment.
linding	Investigators were not blinded to group allocation during experiments.

Reporting for specific materials, systems and methods

We require information from authors about some types of materials, experimental systems and methods used in many studies. Here, indicate whether each material, system or method listed is relevant to your study. If you are not sure if a list item applies to your research, read the appropriate section before selecting a response.

Materials & experimental systems

n/a	Involved in the study	
☐	☒	Antibodies
☐	☒	Eukaryotic cell lines
☒	☐	Palaeontology and archaeology
☐	☒	Animals and other organisms
☒	☐	Human research participants
☒	☐	Clinical data
☒	☐	Dual use research of concern

Methods

n/a	Involved in the study	
☒	☐	ChIP-seq
☐	☒	Flow cytometry
☒	☐	MRI-based neuroimaging

Antibodies

ntibodies used	APC anti-mouse CD11c (Catalog no. 117310), FITC anti-mouse CD80 (Catalog no. 104706), PE anti-mouse CD86 (Catalog no. 105008), FITC anti-mouse CD3 (Catalog no. 100204), APC anti-mouse CD8 (Catalog no. 100712), PE anti-mouse CD4 (Catalog no. 130310), purified anti-mouse CD16/32 (Catalog no. 156604) and Alexa Fluor® 700 anti-mouse CD45 (Catalog no. 103128) were purchased from Biolegend. HMGB1 antibody and cleaved caspase-3 antibody (Catalog no. 9661L) were purchased from Cell Signaling Technology. Secondary antibody Alexa Fluor 488 conjugated donkey anti-rabbit IgG was purchased from Thermo Fisher Scientific (Catalog no. 2045215).
Validation	All antibodies were used in the study according to the profile of manufacturers. Antibody validation for immunofluorescence or flow cytometry was validated by the supplier and confirmed in Figure 3, 5, 6 and Supplementary Figures 17, 18, 22, 23, 24, 26, 27 and 28.

Eukaryotic cell lines

Policy information about cell lines

Cell line source(s)	4T1 murine mammary carcinoma cell line; AML 12 murine liver hepatocyte cell line
Authentication	These cell lines were authenticated by the supplier using STR analysis.
Mycoplasma contamination	No contamination was detected by the supplier using Hoechst DNA stain method, agar culture method, PCR-based assay.
Commonly misidentified lines (See ICLAC register)	These cell lines that we used were not listed in commonly misidentified lines in ICLAC Register.

Animals and other organisms

Policy information about studies involving animals; ARRIVE guidelines recommended for reporting animal research

aboratory animals	Animal experiments were performed in compliance with Guidelines for Care and Use of Laboratory Animals of the Nanyang Technological University-Institutional Animal Care and Use Committee (NTU-IACUC) and approved by the Institutional Animal Care and Use Committee (IACUC) for Animal Experiment, Singapore.

Wild animals	Irrelevant to experiments.
Field-collected samples	Irrelevant to experiments.
Ethics oversight	No ethical issues.

Note that full information on the approval of the study protocol must also be provided in the manuscript.

Flow Cytometry

Plots

Confirm that:

- [ ] The axis labels state the marker and fluorochrome used (e.g. CD4-FITC).
- [x] The axis scales are clearly visible. Include numbers along axes only for bottom left plot of group (a 'group' is an analysis of identical markers).
- [x] All plots are contour plots with outliers or pseudocolor plots.
- [x] A numerical value for number of cells or percentage (with statistics) is provided.

Methodology

Sample preparation	Mice after various therapies were sacrificed, and primary tumors, distant tumors and spleens were harvested and prepared to single cell suspension according to standard protocols. Briefly, tumors were minced into tiny pieces and digested with enzyme cocktail (collagenase type I and IV, 1 mg mL-1) at 37 °C for 1 h. Thereafter, the mixture was gently grinded and filtered through 70 µm cell strainer to obtain single cell suspension. Spleen was placed in 1 × PBS and gently minced with plunger of the syringe. Thereafter, the mixture was filtered through 70 µm cell strainer. Red cells in single cell suspension were removed by eBioscienceTM 1× RBC Lysis Buffer (Invitrogen). In addition, peripheral blood mononuclear cells were isolated by density gradient centrifugation of blood with Histopaque® 1077 (400 ×g, 30 min). For the analysis of CTL and helper T cell, the collected cells were blocked with anti-mouse CD16/32 and stained with Alexa Fluor® 700 anti-mouse CD45, Live/DeadTM Fixable Blue Dead Cell Stain, FITC anti-mouse CD3, APC anti-mouse CD8 and PE anti-mouse CD4 following the vendor's protocols. Thereafter, the stained cells were analysed by flow cytometry.
Instrument	Fortessa X20 (BD Biosciences)
Software	FACS Diva and FlowJo v10
Cell population abundance	No cell sorting was performed.
Gating strategy	Briefly, single cells were selected by FSC and SSC plots. Live cells were selected as defined by Live Dead Stain-negativity. Leukcocytes were gated by CD45+ cells. Gating strategies were indicated in detail in Supplementary Figure 17.

- [x] Tick this box to confirm that a figure exemplifying the gating strategy is provided in the Supplementary Information.
